# Altered oral microbiome in Sudanese Toombak smokeless tobacco users carries a newly emerging risk of squamous cell carcinoma development and progression

**DOI:** 10.1038/s41598-023-32892-y

**Published:** 2023-04-24

**Authors:** Amel Sami, Imad Elimairi, C. Anthony Ryan, Catherine Stanton, Dhrati Patangia, R. Paul Ross

**Affiliations:** 1 APC Microbiome Ireland, School of Microbiology, University College Cork, Cork, T12 YN60 Ireland; 2 Teagasc Food Research Centre, Moorepark, Fermoy, Cork, P61 C996 Ireland; 3 Department of Oral and Maxillofacial Surgery and Oral Medicine, Faculty of Dentistry, National Ribat University, Nile street, 1111 Khartoum, Sudan; 4 Department of Paediatrics and Child Health, University College Cork, Cork, T12 DFK4 Ireland

**Keywords:** Metagenomics, Oral cancer

## Abstract

There are an estimated 6–10 million smokeless tobacco (Toombak) users in Sudan, the majority being males. Toombak is known to be a carcinogenic product that is likely to modify the oral microbiome spatiality into a high-risk potential for the development and progression of oral cancer, but previous studies are lacking in this field. Here, we endeavour for the first time the exploration of the oral microbiome in key mucosal areas of the oral cavity and assess the microbiome variations in premalignant and oral squamous cell carcinoma (OSCC) samples from both users and non-users of Toombak. 16S rRNA sequencing was performed on DNA obtained from pooled saliva, oral mucosa and supragingival plaque from 78 Sudanese users and non-users of Toombak, aged between 20 and 70 years. In 32 of the pooled saliva samples, the mycobiome (fungal) environment was analysed through ITS sequencing. Then, 46 formalin-fixed paraffin-embedded samples of premalignant and OSCC samples were collected, and their associated microbiomes sequenced. The oral Sudanese microbiome was found to be enriched in *Streptococcaceae*, but *Staphylococcaceae* were significantly more abundant amongst Toombak users. Genera enriched in the oral cavity of Toombak users included *Corynebacterium_1* and *Cardiobacterium* while in non-users, *Prevotella, Lactobacillus* and *Bifidobacterium* were prominent. *Aspergillus* was the most abundant fungus in the mouths of Toombak users with a marked loss of *Candida.* The genus *Corynebacterium_1* was abundant in the buccal, floor of the mouth and saliva microbiomes as well as in oral cancer samples from Toombak users indicating a possible role for this genus in the early stages of oral cancer development. An oral cancer microbiome that favours poor survival and metastasis in those who use Toombak also emerged that includes the genera *Stenotrophomonas* and *Schlegelella*. Those utilising Toombak carry an altered oral microbiome that may be an additional risk factor for this products carcinogenicity to the oral structures. These significant microbiome modulations are a newly emerging key driving factor in oral cancer development and progression in Toombak users while it is also shown that Toombak users carry an oral cancer microbiome that may increase the potential for a poorer prognosis.

## Introduction

Smokeless tobaccos harbour bacterial and fungal entities that allow for microorganisms to reside in the oral cavity and their mucosal surfaces which are not normal of the typical oral microbiota. These oral mucosal surfaces are further impacted by salivary pH, flow of saliva and oral hygiene and along with smokeless tobacco use can have a varying impact on the localised keratinised and non-keratinised tissue.

An example of a smokeless tobacco is ‘Toombak’, used predominantly by Sudanese males that is produced from the plant, the *Nicotiana Rustica*. Leaves are harvested, subjected to fermentation and sodium bicarbonate is added to improve the taste and bioavailability of nicotine in the final product. Through these processes, Toombak has been found to carry potently elevated quantities of carcinogenic compounds that include but are not limited to tobacco-specific nitrosamines, formaldehyde, and acetaldehyde^1,2^. Iron is also found to be abundant in Toombak and may be involved in tumour development including oral squamous cell carcinoma (OSCC)^3,4^.

Indeed, OSCC is a disease that in Sudan has been primarily attributed to Toombak use^5^. The oral microbiome of smokeless tobacco users has been shown to be modelled into a dysbiotic state as early as 4 weeks from tobacco initiation^6,7^. Smokeless tobacco use can lead to histological changes in the oral epithelium within just 2 days of its use, causing inflammation and ulceration^8,9^. The roughened texture of smokeless tobacco products, including Toombak is also a supporting factor in the survival of many of the microorganisms found in these products.

Furthermore, smokeless tobacco use has been shown to modify the immune response through the elevation of interleukins 1 and 2 and the reduction of macrophages, interferon γ, and interleukin 10 in local smokeless tobacco placement sites^8^. Smokeless tobacco may further activate the oncogenic RAS gene and help in the upregulation of the oxidative stress pathways; ASK1, JNK 1 and 2 and p38^9^. Toombak users who develop OSCC were found to have increased p53 mutations, novel mutations in exons 5,6 and 7^10^ and an increased expression in keratins 13,14 and 19, that relate to abnormal proliferation and maturation of keratinocytes^11^.

The relationship between the oral microbiome and cancer development has in the last decade been continuously interrogated, albeit with a narrower or absent insight from developing countries^12^. Culture studies from Sudan have highlighted enriched *Bacillus* growth from the buccal mucosa of Toombak users^6^. On the other hand, Mohamed et al. (2021) identified *Malassezia* as a favourable predictor of survival amongst Sudanese OSCC patients^13^.

This study serves to analyse the oral cavity microbial spatiality of the Sudanese, including smokeless tobacco users (a high risk cohort for oral cancer development) through metagenomics sequencing approaches while also helping to understand the implications that Toombak use brings post-OSCC development.

## Material and methods

### Sample collection

Ethical approval was first sought and obtained from the Sudanese ethics committee at National Ribat University Sudan and the Cork Ethics Committee, Cork, Ireland. All experiments were performed in accordance with relevant names guidelines and regulations. Informed consent was obtained from all participants.

Inclusion criteria included those participants with good oral care, inactive periodontal disease, a controlled caries mouth and absence of any other dental infection. Following consent, participants did not eat or drink for at least 1 h before sample collection. In the smokeless tobacco group, oral swab samples were obtained at least 1 h after disregarding the most recent Toombak dip. Exclusion criteria included local conditions such as those with active periodontal disease, caries and periapical infection and systemic factors such as recent antibiotic use (< 3 months) and unstabilised conditions.

We achieved the collection of 72 pooled saliva, 71 supragingival plaque and 272 oral mucosal swab samples from 78 Sudanese participants. These were further categorised into 47 Toombak smokeless tobacco users and 31 non-users aged 20–70 years of age. While all Toombak users were male, in the non-user group, 18 participants were female. Pooled saliva was collected by asking the patient to remain seated, holding a sterile disposable polypropylene Falcon® (110 ml) container and up to 5 ml of saliva was pooled. Oral mucosal swab samples were collected by applying Puritan® Diagnostic Swabs to the required mucosal location with gentle rubbing for 10 s.

**Figure 1:**
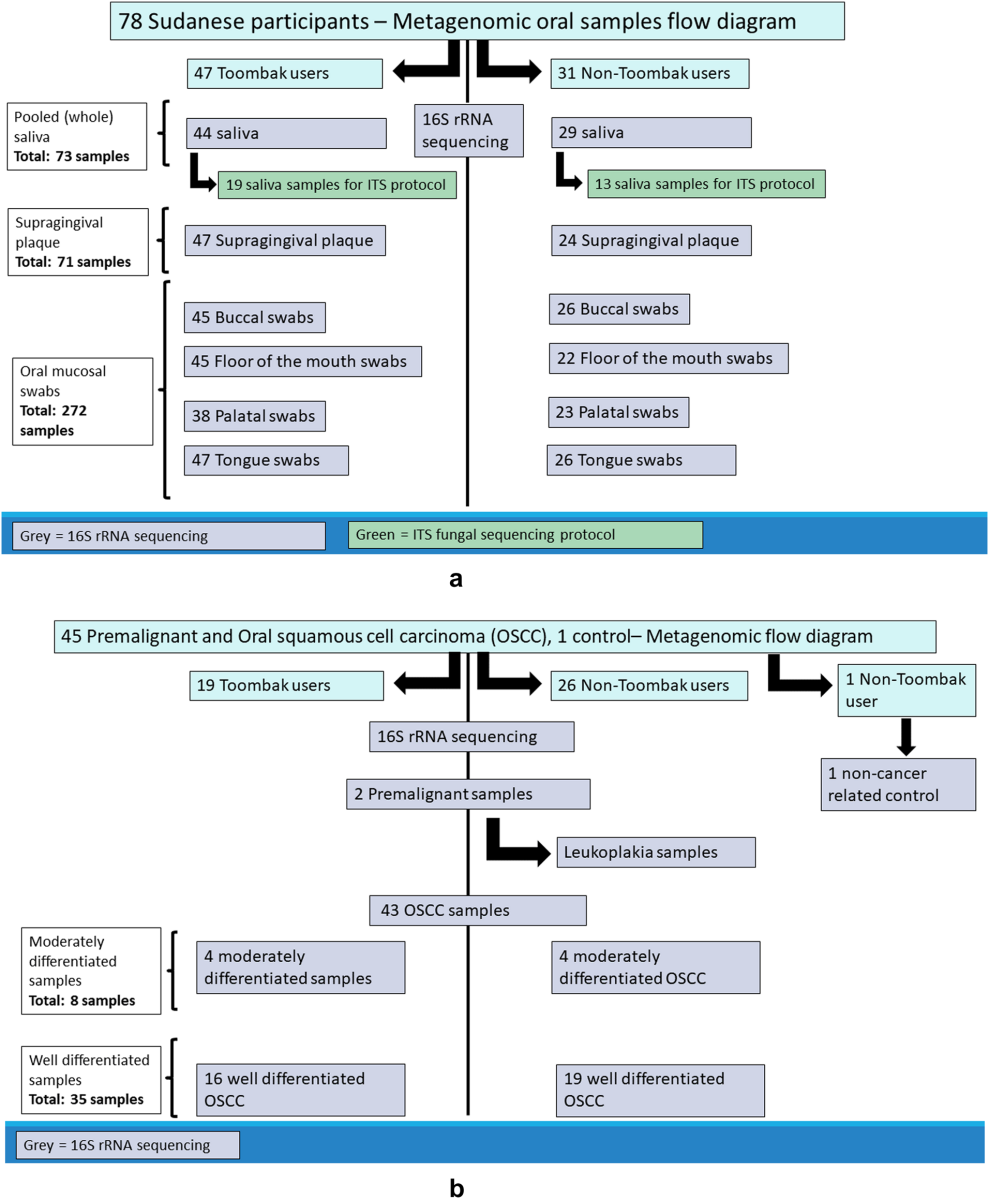
(**a**) 78 Sudanese participants – Metagenomic oral samples flow diagram. Participant samples were categorised into saliva, supragingival plaque and oral mucosa swabs. (**b**) Premalignant and oral squamous cell carcinoma Metagenomic flow diagram. Samples were achieved from formalin fixed paraffin embedded tissues of premalignant, oral cancer and non – cancerous (control) sample.

Supragingival plaque samples were obtained using a sterile probe which was stored in Puritan® Diagnostic Swabs for upstream analysis. The mucosal swabs were collected from two keratinised (dorsum tongue and hard palate) and two non-keratinised locations (floor of the mouth and buccal mucosa). Seventy-one buccal swabs (from 26 non-users and 45 Toombak users), 67 floors of the mouth swabs (from 22 non-users and 45 Toombak users), 61 hard palate swabs (from 23 non-users and 38 Toombak users), and 73 dorsum tongue swabs (from 26 non-users and 47 Toombak users) were obtained. Figure 1a summarises the metagenomic oral sample collection in this study.

### Storage and transfer

Samples were stored in an iced cooler transport box and transferred to a—80 °C freezer in the laboratory at National Ribat University, Sudan. Finally, samples were shipped to Cork, Ireland on dry ice.

### Collection of formalin-fixed paraffin-embedded oral cancer and premalignant tissue samples

Forty-six formalin-fixed paraffin-embedded samples were collected (from 43 OSCC, two premalignant lesions (Leukoplakia) and one non- premalignant condition). The OSCC samples were further delineated as eight moderately differentiated and 35 well differentiated OSCC samples. Samples were sourced from 29 males and 17 females, aged between 20 and 70 years of age. Twenty-six samples were from non-Toombak users and 19 were from Toombak users. Figure 1b summarises the metagenomic premalignant and OSCC samples collection flow obtained in this study. To yield optimum DNA from paraffin-embedded samples, we used 10 μm thickness to slice sections with the first 2–3 sections being discarded. Eight sections were used for each sample. All formalin fixed paraffin embedded tissue samples were no longer than 3 months old from fresh sample preparation and samples were collected pre-cancer treatment. This time length was kept consistent to exclude any bias that could be introduced by time variations. Sections were immediately placed in sterile 2 ml Eppendorf tubes.

### DNA extraction of pooled saliva, mucosal swab, and plaque samples

DNA extraction of samples was achieved by the DNeasy Powersoil Pro Kit (QIAGEN®, Hilden, Germany). Eluted DNA was transferred to sterile Eppendorf tubes and frozen at − 80 °C for further analysis. The mucosal swabs and plaque were first cut using a sterile scissor which allowed placement and dissolvement of samples in DNA extraction tubes. Swabs were removed after vortexing during the first stage of DNA extraction.

### DNA extraction of formalin-fixed paraffin-embedded oral cancer and premalignant tissue samples

Here, QIAamp® DNA formalin fixed paraffin embedded (FFPE) tissue kit instructions were followed. Briefly, in a microcentrifuge tube, xylene was placed with each sample to remove the paraffin and centrifuged at full speed for two min, after which 1 ml of ethanol was added to remove the xylene. Samples were vortexed and centrifuged to remove residual ethanol, maintaining the pellet. Tubes were incubated at 37 °C, resuspended in 180μl tissue lysis buffer and 20 μl proteinase K was added to overcome the inhibitory effects caused by formalin crosslinking of nucleic acids. All buffers were prepared according to the manufacturer’s instructions and equilibrated to room temperature before protocol initiation. Samples were incubated at 56 °C for 1 h and 90 °C for another hour. The incubation in the latter temperature reverses the modification of nucleic acids by formaldehyde. DNA extraction was continued by adding 200 μl lysis buffer and vortexing until reaching a homogenous lysate. QIAamp® MinElute columns were used to allow for the purification of high-quality DNA from the lysate and centrifuged at 6000× g (8000 rpm) for 1 min. This step was repeated until all lysates had passed through the columns, and the QIAamp® MinElute columns were empty. 500 μl of wash buffer was added and samples were centrifuged for 1 min at 6000× g (8000 rpm). Columns were placed in a clean 2 ml collection tube, the flow-through discarded and centrifuged at high speed; 20,000× g (14,000 rpm) for 3 min to ensure all ethanol was removed. Finally, columns were placed in clean 1.5 ml microcentrifuge tubes, incubated at room temperature for 5 min in 50 μl buffer and then centrifuged for 1 min to yield the final product at 20,000× g (14,000 rpm). Samples were stored at − 80 °C for upstream analysis.

### Amplicon sequencing

The Illumina Metagenomic Sequencing Library Preparation protocol for 16S rRNA gene library preparation was followed. Briefly, PCR was performed on DNA extracted samples using AMPure XP beads (Beckman Coulter), universal forward primers and reverse primers with overhang adapters, following standard IUPAC nucleotide nomenclature, to target the 16S V3–V4 hypervariable regions. PCR was then performed in a 50 μL reaction mixture containing 10 ng of template DNA and 2 × KAPA HiFi HotStart Ready Mix. The following thermal cycling conditions were used: 3 min of initial denaturation at 95 °C; 30 cycles each of denaturation at 95 °C for 30 s, annealing at 55 °C for 30 s, and elongation at 72 °C for 30 s and the last step at 72 °C for 5 min.

PCR clean-up was achieved with AMPure XP beads (Beckman Coulter) to purify the 16S V3 and V4 amplicon from free primers and primer dimer species. Electrophoresis was performed on the PCR-amplified products using 1.5% agarose gel with Invitrogen DNA loading dye (ThermoFisher Scientific) to verify the expected size at 550 base pairs. The gel was then visualised under 300 nm ultraviolet light. Index PCR was then continued by attaching dual indices, and Illumina sequencing adapters using Nextera XT index kits, and the following PCR thermal cycling conditions were used: 3 min of initial denaturation at 95 °C; 8 cycles each of denaturation at 95 °C for 30 s, annealing at 55 °C for 30 s, and elongation at 72 °C for 30 s; the last step at 72 °C for 5 min. AMPure XP beads (Beckman Coulter) were utilised for final clean-up, and the final library was quantified using the Qubit® 2.0 Fluorometer and the Qubit dsDNA HS Assay kit (ThermoFisher Scientific). Samples were then normalised, with the final library run on an Agilent high sensitivity chip and quantified by qPCR using the KAPA Illumina Library quantification kit.

In addition, the saliva samples of 13 non-users and 19 Toombak users were processed for ITS gene sequencing. This was prepared with a similar method to the 16S rRNA gene protocol and followed the methods described by Walsh et al. ^14^. The primers were specific to the ITS1-ITS2 regions of the ITS gene and included the Illuminas overhang adaptors (ITSF1 primer 5′ TCGTCGGCAGCGTCAGATGTGTATAAGAGACAGCTTGGTCATTTAGAGGAAGTAA 3′ and ITS2 primer 5′GTCTCGTGGGCTCGGAGATGTGTATAAGAGACAGGCTGCGTTCTTCATCGATGC 3′).

For bacterial analysis, PCR products were sequenced using the V3-V4 regions of the 16S rRNA gene on an Illumina MiSeq device two × 300 platforms (Illumina, Inc. San Diego) according to the manufacturer's instructions. The 300 base pair paired-ends FastQ product generated from 16S sequencing was merged using FLASH (fast length adjustment of short reads) using default parameters^15^. QIIME’s split_libraries_fastq.py script was used for demultiplexing and filtering the fastq sequence data. Further quality filtering was performed using the USEARCH analysis tool. Single unique reads were removed followed by denoising and chimera removal. Grouping into operational taxonomic units (OTUs) at 97% similarity was performed using USEARCH v7 64bit^16^. OTUs were aligned using Pynast (PyNAST: python nearest alignment space termination; a flexible tool for aligning sequences to a template alignment^17^. Further taxonomic ranks were set using the Basic Local Alignment Search Tool (BLAST) against the SILVA SSURef database release 132^18^. Data were processed using Microbiome Analyst CA and Calypso version 8.84 with data filtering (removal of low abundance features less than 10%). Data were normalised utilising total sum scaling (TSS). The U.S National Library of Medicine, national centre for biotechnology information, was then accessed to assign species identity to those FASTA sequences with > 98% percentage homogeneity by using the BLAST® tool for bacterial 16S rRNA sequencing database^19^.

For fungal analysis, the resulting sequencing reads were variable in length and were processed using DADA2 (v1.18.0) in R, following DADA2 pipeline workflow for ITS sequences^20,21^. The reads were filtered to remove sequences with ambiguous ‘N’ bases. This was followed by primer removal from the reads using Cutadapt (v3.5) to remove primers as described in the DADA2 ITS pipeline workflow^22^. The quality of the reads was inspected, and the reads were further filtered and trimmed to retain reads that were a minimum of 50 base pair long with default parameters. Dereplicating and merging of paired-end reads was done following the workflow, following which chimeras were removed and taxonomy was assigned to the resulting ASVs (Amplicon Sequence Variants) using the UNITE ITS database. The resulting ASV table was used for further analysis with Microbiome Analyst.

Data were not rarefied but normalised utilising total sum scaling (TSS). Calypso version 8.84 and Microbiome analyst CA^23^ were used for the statistical and interpretational metagenomic data.

## Results and discussion

For the plaque and oral mucosal samples, a total of 1344 observations were encountered with a minimum read of 8.0 (controls) and a maximum read of 2,008,537.0.

### Alpha and beta diversity

Samples were grouped by mucosal location (buccal cheek, floor of the mouth, hard palate and dorsum tongue) and status of Toombak use (healthy = non-user, user = Toombak user). We found that mucosal location in the oral cavity and keratinisation status is a stronger predictor of microbiome variation compared to Toombak use. Non-keratinised mucosal locations (buccal and floor of the mouth) had similar patterns in microbiome diversity while keratinised locations also harboured close resemblances (palate and tongue). However, utilising Toombak alters the oral microbiome regardless of optimum oral health.

**Figure 2:**
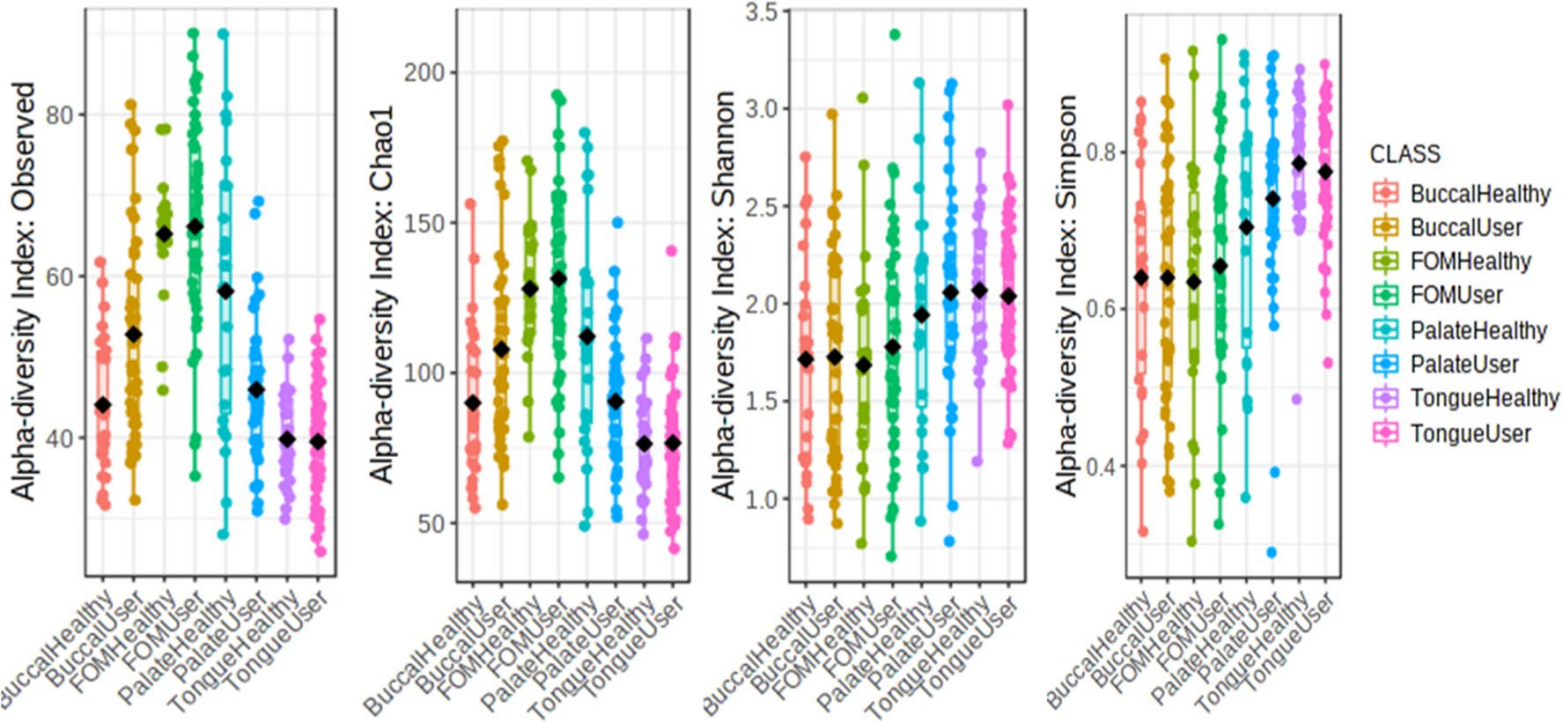
**α** diversity variation between four oral mucosal locations (buccal, floor of mouth (FOM), palate and tongue) in users and non-users of Toombak. 2. Shannon index (*p* = 0.00076), Richness or Observed (*p* = 3.6849e−34) and Simpson’s index (*p* = 3.3603e−08) were all significant between groups. Richness was found to be lowest for tongue microbiome but similar in the remaining locations. The tongue and palatal mucosa (Simpson’s index community diversity closest to 1) show the closest clustering.

Four alpha diversity measures, Chao1, Shannon and Simpson index, and richness were assessed (Fig. 2). Alpha diversity was found to be significantly varied between groups. Chao 1, a measure of variation between the rare or unobserved species, showed that the tongue mucosa had the lowest Chao 1 diversity in both users and non-users of Toombak. In the palatal mucosa, those who used Toombak, however, had lower Chao 1 indices compared to non-users. Shannon indices were more distinct by location rather than Toombak use (*p* = 0.00076). Simpson index showed a higher but narrower tongue and palatal microbiome compared to the remaining groups (Simpson’s index community diversity closest to 1). Richness (*p* = 3.6849e−34) was highest on the floor of the mouth mucosa but lowest on the tongue mucosa. The median richness of the buccal mucosa microbiome was markedly lower for non-users compared to users of smokeless tobacco.

**Figure 3:**
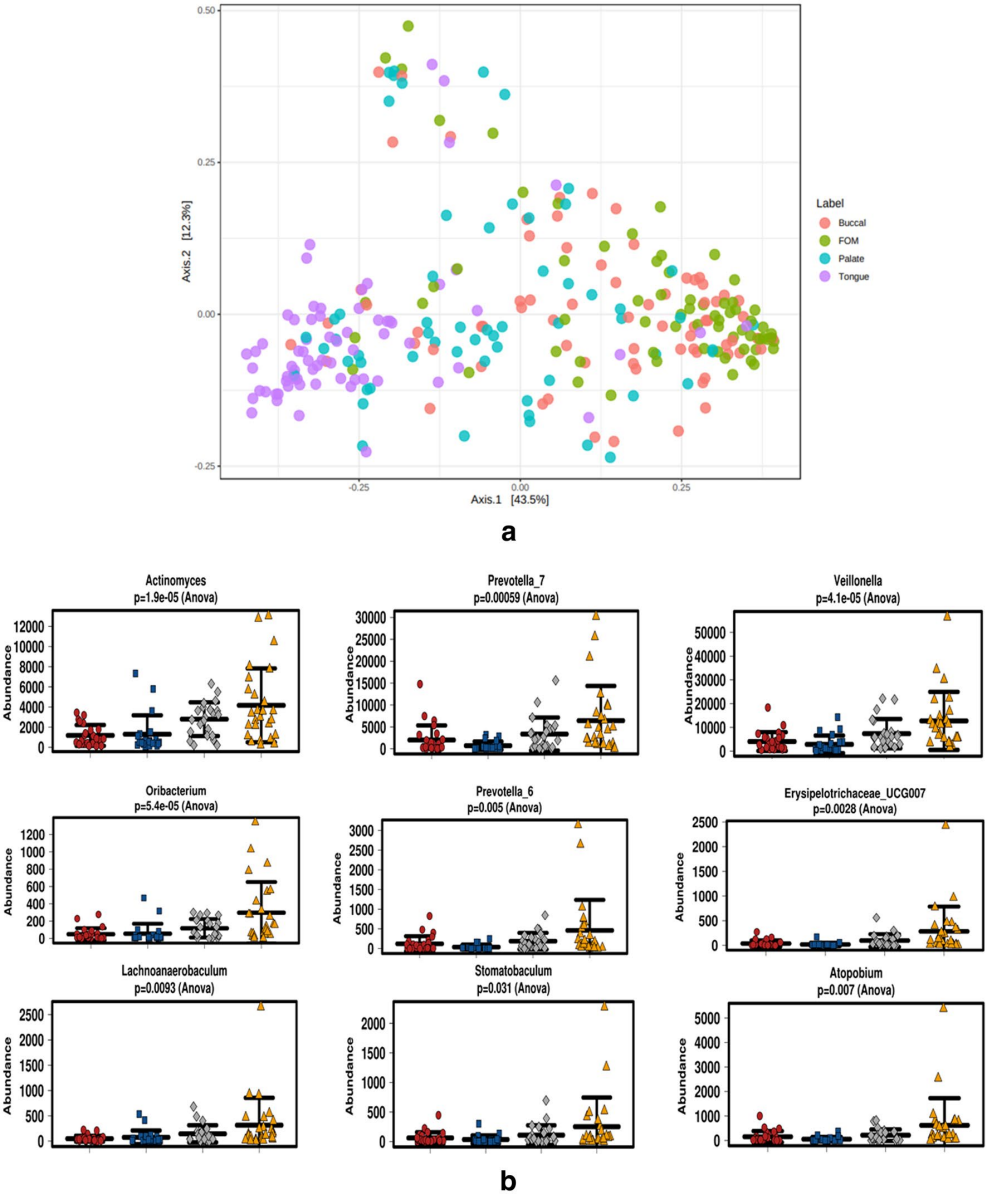

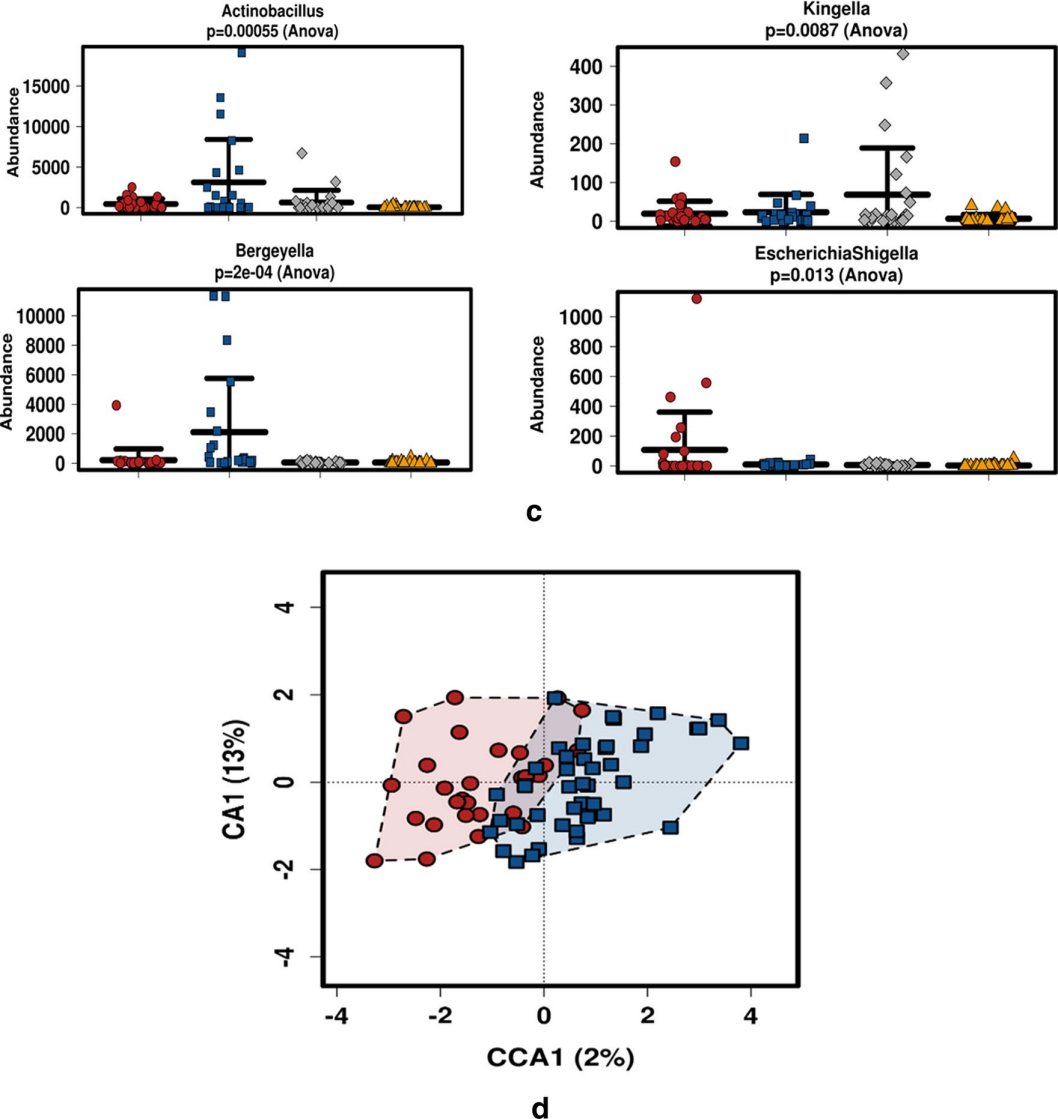
(**a**) β diversity between the oral mucosal regions. Significant β diversity between the oral mucosal regions (*p*-value < 0.001). R^2^ = 0.22. The tongue microbiome (purple) is most distinct. Non-keratinised buccal and floor of the mouth mucosa are the most clustered together while the palatal microbiome is centrally bridged. (**b**) Tongue microbiome abundances amongst the oral Sudanese microbiomes. Red = buccal cheek, blue = floor of the mouth, grey = hard palate and gold = dorsum tongue. *Actinomyces, Prevotella, Veillonella* are amongst the most significantly abundant genera of tongue microbiome. (**c**) Abundant genera in the other mucosal locations. Red = buccal, blue = floor of the mouth, grey = palate, and gold = dorsum tongue. *Actinobacillus* and *Bergeyella* are most abundant in the floor of the mouth, *Kingella* in the palate and *Shigella* in the buccal mucosa. (**d**) Canonical correspondence analysis (CCA) plot of users (blue) and non-users (red) of Toombak. Oral health was utilised as the known gradient in minimising matrix errors in OTU–oral health relationships. Accounting for oral health, Toombak harboured oral microbiome differences that could be appreciated compared to non-users.

Beta diversity (Fig. 3a) between the oral mucosal regions was significantly distinct (*p* < 0.001 R^2^ = 0.22), particularly between the dorsum tongue and the buccal/floor of the mouth, which were found to cluster together. The palatal microbiome was ‘sandwiched’ between the microbiome of the other mucosal environments. Such diversity could be due to the genera and species composition found in the various mucosal habitats. The tongue microbiome harboured an abundance of *Actinomyces, Prevotella_6, Prevotella_7, Veillonella, Oribacterium, Erysipelotrichaceae_UCG007, Lachnoanaerobaculum, Stomatobaculum* and *Atopobium* (Fig. 3b). The floor of the mouth mucosa harboured increases in the genera *Actinobacillus* and *Bergeyella*, while the palatal mucosa was increased in *Kingella*, after which the buccal cheek mucosa was found to be significantly increased in the genus *Shigella* (Fig. 3C).

We further used canonical correspondence analysis (CCA) as a multivariate error-reducing technique analysing the abundance of the oral microbiome within users and non-users of Toombak on the known gradient oral health minimising matrix errors in OTU-oral health environmental relationships^24^. CCA plotting (Fig. 3d) showed that after accounting for oral health, Toombak users and non-users of Toombak still harboured distinct oral microbiome variations.

### Pro-carcinogenic phyla and premalignant associated classes of bacteria are abundant in the saliva of Toombak users

**Figure 4:**
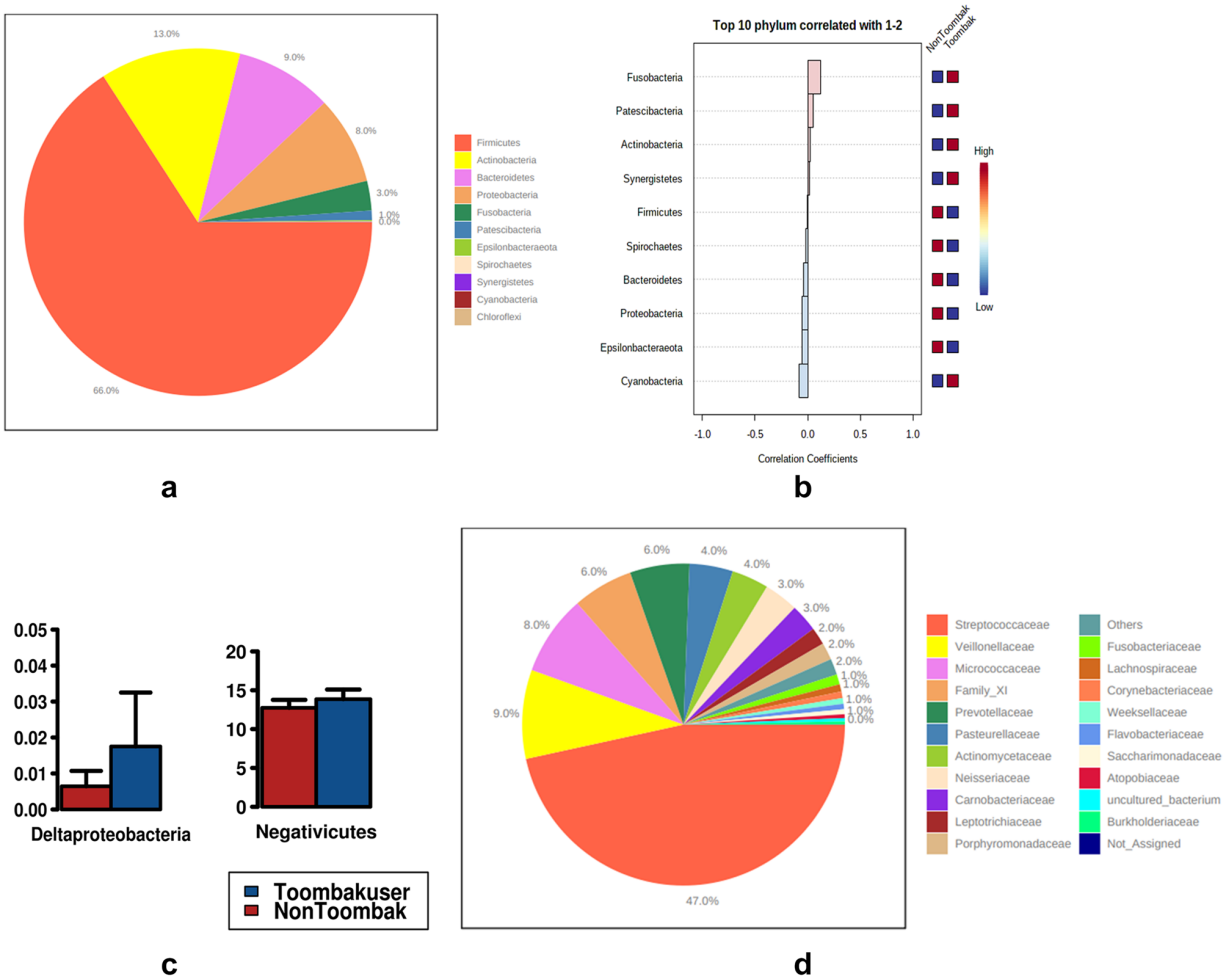
(**a**) Pie chart percentage composition of phyla from pooled saliva of both users and non-users of Toombak. *Firmicutes* (66%), *Actinobacteria* (13%), *Bacteroidetes* (9%), *Proteobacteria* (8%) and *Fusobacteria* (3%) are the most relatively abundant phyla. (**b**) Correlation plot of ten most abundant phyla in the Sudanese oral cavity of users and non-users of Toombak. *Fusobacteria* are the most correlated with Toombak use compared to *Cyanobacteria* in non-users. (**c**) Log-bar values of class composition of pooled saliva of both users and non-users of Toombak. Increased abundances in the classes *Deltaproteobacteria* and a slight increase in *Negativicutes* were found among Toombak users. (**d**) Pie chart percentage composition of families from pooled saliva of both users and non-users of Toombak. Streptococcaceae (47%), *Veillonellaceae* (9%) and *Micrococcaceae* (8%) were the most relatively abundant while *Staphylococcaceae* were the most significantly differentiating family between users and non-users of Toombak with a *p*-adjusted value of 0.037.

The most relatively abundant phyla in pooled saliva were found to be *Firmicutes* (66%), *Actinobacteria* (13%), *Bacteroidetes* (9%), *Proteobacteria* (8%) and *Fusobacteria* (3%) (Fig. 4a). We further utilised correlation plotting to visualise the top ten phyla varied in abundance between the saliva of users and non-users of Toombak. Here, the phyla *Fusobacteria* and *Patescibacteria* (also known as *Candidate Phyla Radiation*) were the most correlated for Toombak use while *Cyanobacteria* were associated with non-users of Toombak (Fig. 4b). Both *Fusobacteria* and *Patescibacteria* have been shown to be significantly enriched in various forms of gastric cancer while *Fusobacteria* in particular have been shown to promote cancer progression and the invasion of cancer into surrounding tissue^25^. They are said to be late colonisers in healthy individuals and their abundance amongst Toombak users could have a role to play in the early developmental changes of OSCC^26^. This is likely due to *Fusobacteria* possessing FadA proteins that are associated with the attachment, invasion, and adherence of cancer cells^27^. *Gracilibacteria* were also found to be abundant amongst Toombak users. These are part of the *Patescibacteria* group that contain unique antimicrobial peptides and heavy metal (nickel) resistance pathways which may make them more tolerant to the heavy metal content of Toombak^28,29^. Classes of bacteria associated with premalignancy were found to be increased in Toombak users. *Negativicutes* amongst Toombak users may predispose them to the development of oral leukoplakia, a premalignant condition^30,31^. *Deltaproteobacteria*, a class of diverse sulphate-reducing bacteria can be pathogenic and cancer-promoting due to their ability to produce hydrogen sulphide^32^,^33^ (Fig. 4c) and were found to be high in Toombak users.

### Microbial family and genera discrepancies between saliva and plaque of users and non-users of Toombak

*Staphylococcaceae* significantly differentiated between users and non-users of Toombak (q = 0.037). At family level, the saliva of users and non-users of Toombak indicated similar relative profiles of *Streptococcaceae* (47%), *Veillonellaceae* (9%) and *Micrococcaceae* (8%) (Fig. 4d). Sixty-five core microbiome genera were identified in the saliva of users and non-users of Toombak. Three genera, however, *Streptobacillus, Shuttleworthia* and *Eubacterium yuri* group, were unique to non-users of smokeless tobacco. In comparison, four genera, *Fretibacterium, Filifactor, Corynebacterium_1* and *Alysiella*, were unique to Toombak users.

**Figure 5:**
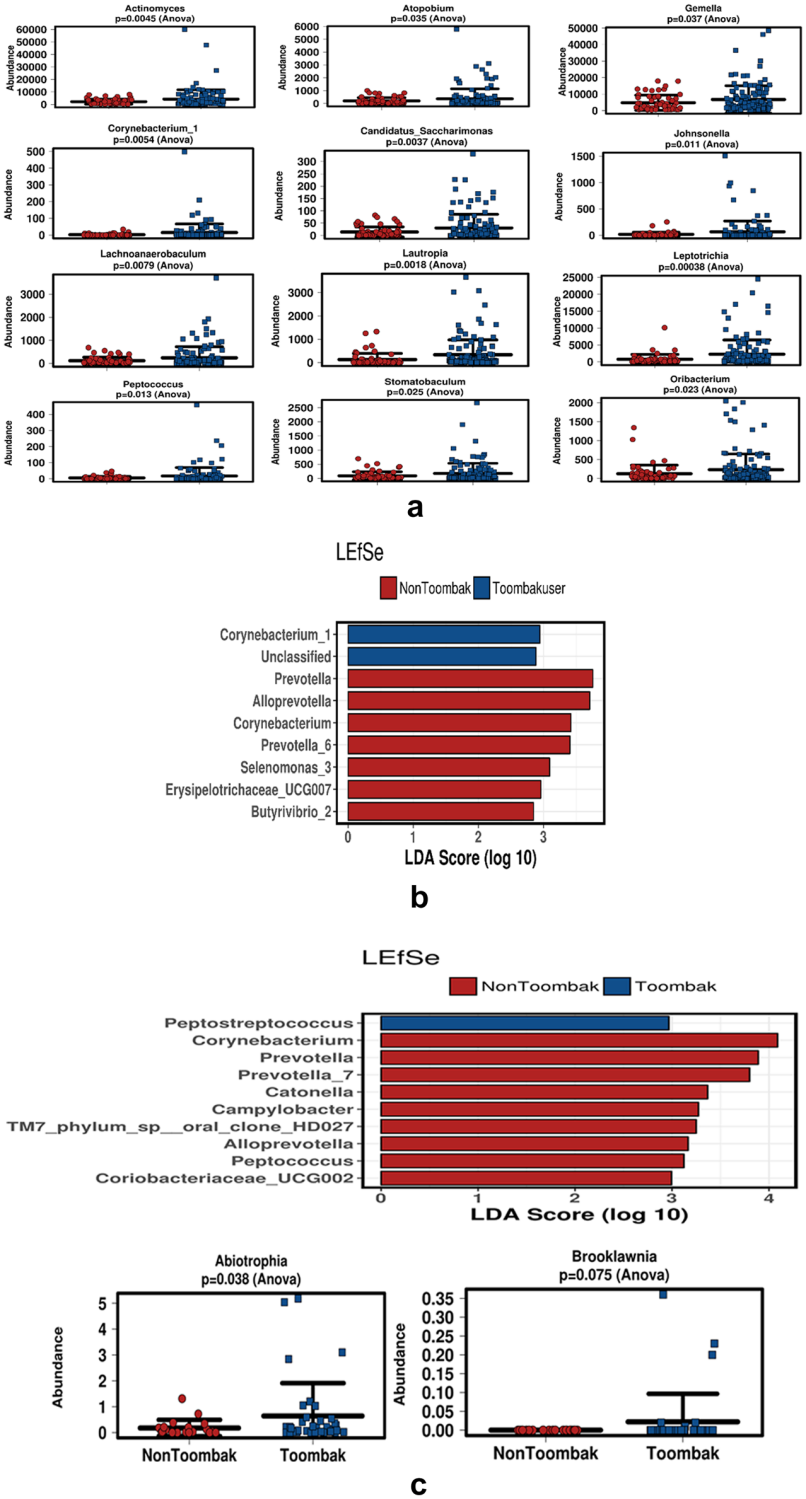
(**a**) The most abundant genera with *p*-value significance amongst Toombak users (blue) and non-users (red). One way Anova variations between significant genera in Toombak use and non-Toombak use. *Actinomyces* (*p* = 0.0045), *Corynebacterium_1* (*p* = 0.0054), *Lachnoanerobaculum* (*p* = 0.0079), *Lautropia* (*p* = 0.0018), *Atopobium* (*p* = 0.035), *Gemella* (*p* = 0.037), *Johnsonella* (*p* = 0.011), *Leptotrichia* (*p* = 0.00038), *Candidatus_Saccharimonas* (*p* = 0.0037), *Peptococcus* (*p* = 0.013), *Ruminococcaceae_UCG014* (*p* = 0.0052) as well as *Oribacterium* (*p* = 0.023) and uncultured bacterium (*p* = 0.025) were more abundant in Toombak users. (**b**) LEfSe plotting on pooled saliva dataset. LEfSe plotting on pooled saliva dataset indicated that *Corynebacterium_1* and unclassified bacterium were discriminant for Toombak use while* Prevotella, Selenomonas*, *Erysipelotrichaceae* and *Butyrivibrio_2* were discriminant for non-users. (**c**) Supragingival microbiome in the Sudanese population and the relative abundances of significant genera in plaque. LEfSe plotting showed that *Peptostreptococcus* was the most distinct genus amongst Toombak users while *Corynebacterium, Prevotella* and *Catonella* were the most distinguishing genera in non-users plaque samples. *Abiotrophia* (*p* = 0.038) and *Brooklawnia* (*p* = 0.075) were on the other hand significantly abundant in the plaque of Toombak users.

The most significantly abundant microbial genera amongst Toombak users are highlighted in Fig. 5a which include *Actinomyces* (*p* = 0.0045), *Corynebacterium_1* (*p* = 0.0054), *Lachnoanerobaculum* (*p* = 0.0079), *Lautropia* (*p* = 0.0018), *Atopobium* (*p* = 0.035), *Gemella* (*p* = 0.037), *Johnsonella* (*p* = 0.011), *Leptotrichia* (*p* = 0.00038), *Candidatus_Saccharimonas* (*p* = 0.0037), *Peptococcus* (*p* = 0.013), *Ruminococcaceae_UCG014* (*p* = 0.0052), *Oribacterium* (*p* = 0.023) and unclassified and uncultured bacterium.

In a study assessing the tongue microbiome of smokeless tobacco users in Saudi Arabia, species of *Actinomyces* and *Oribacterium* were also found to be significantly abundant in smokeless tobacco users^34^ and in smokeless tobacco users from Guam^35^. Elevated levels of *Atopobium* has been found in OSCC biopsies, and *Leptotrichia*, *Gemella* and *Oribacterium* in the saliva of OSCC patients^36^. *Jonhsonella*, found to be abundant in Toombak users, has been highly associated with oral tumour sites indicating specific microbiome dispositional trends in those that utilise Toombak towards oral cancer development^37^.

In a culture-based study from Sudan by Ali et.al. (2014), oral swab samples from long-term smokers showed abundances in *Peptococcus*, a genus also found to be significantly increased in those who develop oral cancer^38^. *Lachnoanaerobaculum* was relatively abundant in the saliva of Toombak users, similar to the findings from other studies^39^. *Lachnoanaerobaculum* was also abundant in smokeless tobacco users as well as oral cancer patients in studies from India^40,41^. This genus has further been found to be significantly increased in a group of pipe or ‘medwakh’ smokers from the United Arab Emirates^42^. Another elevated genus found in Toombak users in this study was *Leptotrichia*, associated with more severe levels of oral epithelial dysplasia^43^ including in those who developed pancreatic cancer^44^. *Leptotrichia* abundance in the oral cavity has been implicated in head and neck cancer^45^ and it was significantly increased in a cohort of heavy smokers in one study from the United Arab Emirates^46^.

Utilising Mann Whitney Kruskal Wallis with *p* adjusted values (q value); two genera were significantly increased in Toombak users; *Corynebacterium_1* (q = 0.0286) and *Staphylococcus* (q = 0.0286) while *Scardovia* was significantly abundant in non-users of Toombak (q = 1.9435e−4). Both *Coryenbacterium_1* and *Staphylococcus*, found to be abundant in the saliva of Toombak users have also been found to be abundant in the Toombak microbiome composition^1^. *Corynebacterium* has been found to be significantly increased in the saliva of smokeless tobacco users from other studies^47,48^. LEfSe plotting on the pooled saliva dataset further indicated *Corynebacterium_1* and unclassified bacterium to be discriminant for Toombak use while *Prevotella, Selenomonas*, *Erysipelotrichaceae* and *Butyrivibrio _2* are discriminant for the saliva of non-users of Toombak (Fig. 5b).

In supragingival plaque, LEfSe plotting showed that *Peptostreptococcus* was the distinct genus amongst Toombak users while the genera *Corynebacterium, Prevotella* and *Catonella* were the most distinguishing genera in plaque of non-users (Fig. 5c). Fifty eight genera were shared between users and non-users of Toombak. *Comamonas* was only found in the plaque of Toombak users while *Abiotrophia* (*p* = 0.038) and *Brooklawnia* (*p* = 0.075) were significantly abundant in Toombak users (Fig. 5c). Non-users of Toombak harboured *Scardovia, Peptococcus, Lactobacillus, Fretibacterium, Catonella* and *Bifidobacterium* in the plaque microbiome composition.

### *Aspergillus* abundance amongst Toombak users likely exposes users to carcinogenic aflatoxin compounds

In this study, we found more than a three-fold enrichment of *Aspergillus* in the oral cavity of Toombak users (78.93%) compared to non-users (21.07%). Aflatoxins produced by *Aspergillus* species can have long-term adverse effects in the causation of cancer^49^. The abundance of *Aspergillus* in the oral cavity is now strongly associated with premalignant conditions such as Lichen planus and thus could predispose Toombak users to developing conditions that favour OSCC development^50^. *Aspergillus* abundance has further been associated with lymph node involvement in OSCC patients from Sudan^13^.

**Figure 6:**
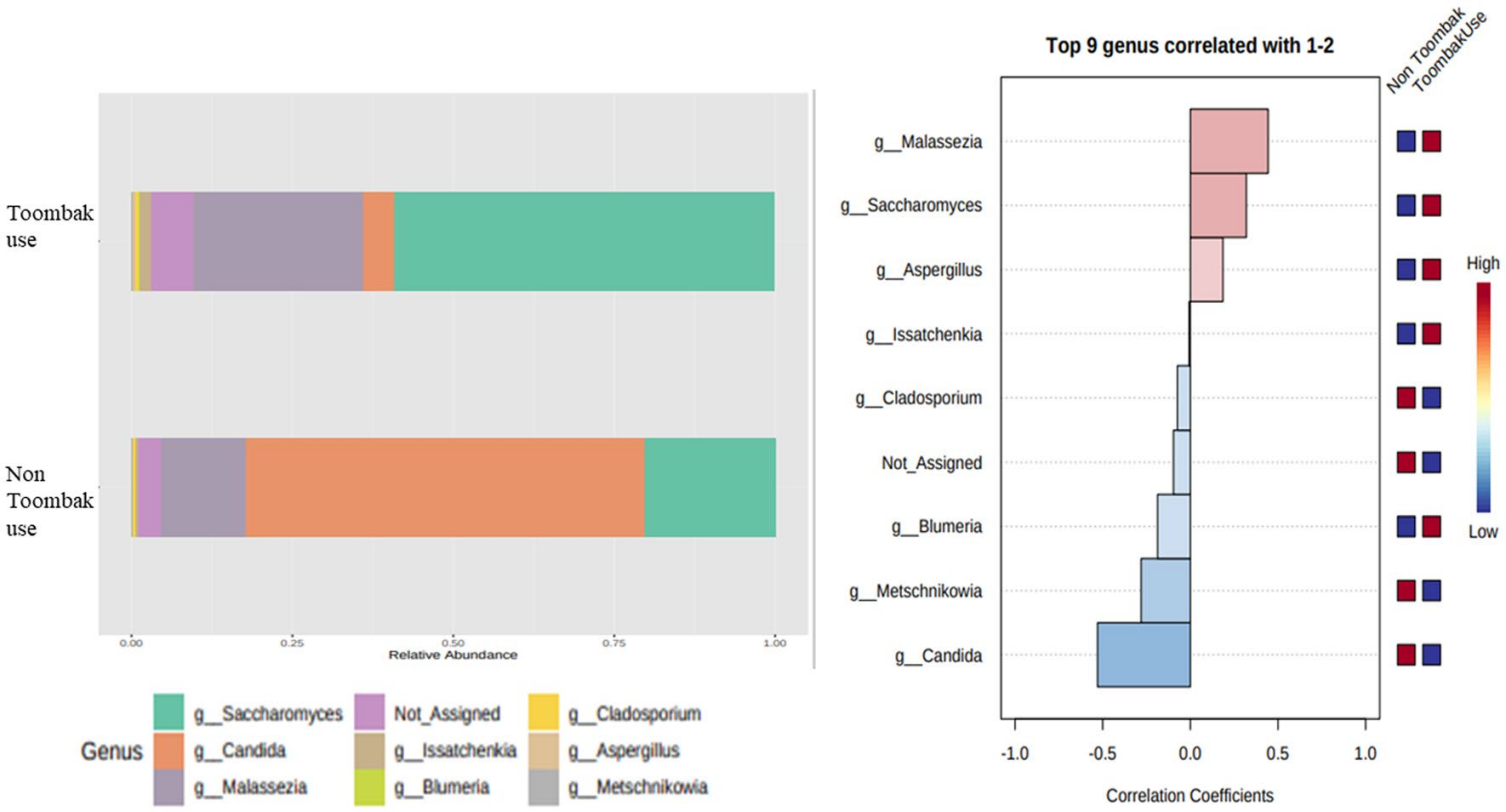
Fungal entities in pooled saliva amongst Sudanese users and non-users of Toombak and correlation differentiators of fungal inhabitance in the oral cavity between Toombak users and non-users. Bar graph abundance (right) highlights marked loss of *Candida* presence in the oral cavity of Toombak users and an increase in *Saccharomyces* and *Aspergillus*. Unassigned species were also abundant amongst Toombak users. Correlation plotting (left) configures abundances in *Malassezia, Saccharomyces* and *Aspergillus* that were correlated positively with Toombak use while *Candida* was the  genus most correlated with non-users of Toombak.

In Toombak users, the mycobiome was found to be abundant in *Blumeria* (61.7%), *Issatchenkia* (61.52%) and *Saccharomyces* (62.11%) compared to non-users (Fig. 6). *Metschnikowia* (36.22%) and *Cladosporium* (65.07%) were lower in abundance amongst Toombak users compared to non-users. *Malassezia* was found to be positively correlated with Toombak use while *Candida* was associated with non-users (Fig. 6). Indeed, there was a marked loss of *Candida* in the oral cavity in those utilising smokeless tobacco (4.33%) compared to non-users (95.67%) (q = 4.758e−4). Interestingly, in a recent study of salivary mycobiome of Sudanese OSCC patients, *Candida* was also found to be reduced in abundance amongst Toombak users^13^. One reason for this could be the inhibitory effects that high nicotine levels (found in Toombak) have on the growth of *Candida* ^51^.

### Fungal species are more virulent with Toombak smokeless tobacco use

*Candida albicans* was found to be the most dominant species amongst non-users, while *Candida tropicalis* was the most abundant species amongst Toombak users. *Candida tropicalis* is one of the most virulent *Candida* species and is known to be resistant to many antifungal medications^52,53^. This species has also been previously isolated from OSCC samples^54^. *Malassezia restricta* was found to be more abundant amongst Toombak users (63.02%) compared to non-users (36.98%) where q = 0.046. An abundance of *Malassezia* may be linked to better survival rates amongst OSCC patients^13^.

### Toombak use allows for significant microbiome variations throughout the four oral mucosal locations

In four mucosal locations, low abundance features were removed based on 10% prevalence and low variance features were further removed based on 5% standard deviation. For data OTU analysis in both users and non-users of Toombak, 274 OTUs were assessed from the buccal cheek mucosa, 208 OTUs from the dorsum tongue mucosa, 254 OTUs from the floor of the mouth and 271 OTUs were assessed from the hard palatal mucosa. We further evaluated the core microbiome of each of the mucosal locations between users and non-users of Toombak. The buccal cheek mucosa had the highest similarity of core microbiome between users and non-users of Toombak; 126 core genera while the dorsum tongue had the least similarity in core microbiome between users and non-users of Toombak; 42 core genera. 53 core microbiome genera were present on the floor of the mouth and 55 core microbial genera on the hard palate between users and non-users of Toombak.

### The buccal mucosa (inner cheek lining)

**Figure 7:**
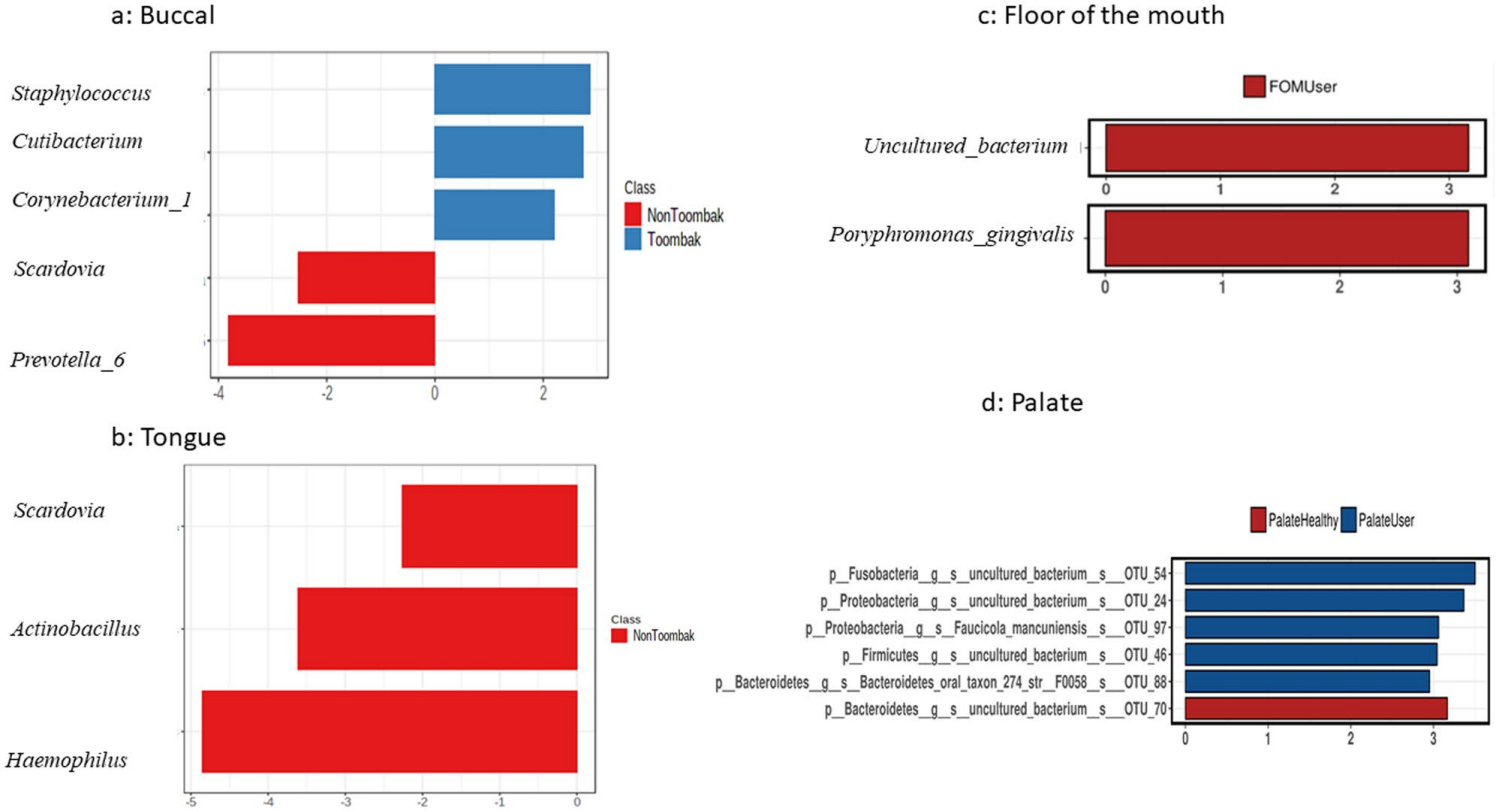
LEfSe plotting of the four oral mucosal locations. (**a**) Buccal cheek microbiome. *Staphylococcus, Corynebacterium_1* and *Cutibacterium* were the most discriminant genera in Toombak users while *Prevotella* and *Scardovia* were found in non-users. (**b**) Dorsum tongue microbiome. *Haemophilus, Actinobacillus,* and *Scardovia* were discriminant in the dorsum tongue of non-users of Toombak. (**c**). The floor of the mouth microbiome. uncultured bacterium (LDA = 3.1) *and Porphyromonas gingivalis* (LDA = 3.09) are the most distinct microbiome components in the floor of the mouth of Toombak users. (**d**) Hard palate microbiome. Uncultured bacterium from the phyla *Fusobacteria* and *Proteobacteria*, and the species, *Faucicola mancuniensis* are observed in Toombak users while uncultured bacterium from the phyla *Bacteroidetes* are discriminant of non-users.

The relative abundance of *Actinobacteria* and *Fusobacteria* were increased in the buccal cheek mucosa of Toombak users compared to non-users of Toombak. *Moraxellaceae* (q = 0.037), *Leptotrichiaceae* (q = 0.019), and *Staphylococcaceae* (q = 0.037) were the most significantly relatively abundant families amongst the buccal microbiome of Toombak users. *Staphylococcaceae* exhibited the highest fold change increase amongst Toombak users (4.259), while *Enterobacteriaceae* (0.037) using Deseq2 coverage were found to be increased amongst non-users of Toombak with a fold change increase of 7.691. *Leptotrichia* (q = 0.029), *Staphylococcus* (q = 0.04) and *Cutibacterium* (q = 0.04) were the most significantly abundant genera amongst the buccal mucosa of Toombak users while LEfSe highlighted *Staphylococcus, Cutibacterium and Corynebacterium_1* (LDA > 2) as the most discriminant genera of the buccal microbiome amongst this group. In the non-users of Toombak, *Shigella* (q = 0.03) and *Prevotella_6* (q = 0.04) were significantly abundant while *Scardovia* and *Prevotella_6* could distinguish the buccal microbiome of non-users (Fig. 7a).

Genera found in abundance in the buccal microbiome of Toombak users may be related to the increased risk of oral tumour development^47^. *Fusobacteria* and the genera *Staphylococcus* and *Corynebacterium_1* are able to reduce nitrate and nitrite to produce carcinogenic tobacco-specific nitrosamine compounds^29^. Studies from Indian smokeless tobacco users have also highlighted an abundance of *Staphylococcus* amongst OSCC smokeless tobacco users^55^. *Cutibacterium* are likely introduced by using fingers in the oral cavity during Toombak placement^1^. Biofilm formation is enhanced with smokeless tobacco use which can improve epithelial adherence of *Staphylococcus*^46^. In addition, oral *Staphylococcus* abundance amongst Toombak users may be linked to an increased fungal load in the oral cavity^56^ likely through chemical interplay involving metabolites, quorum sensing effectors and stable biofilm development^57^. Oral *Staphylococcus* abundance found in Toombak users may be a reservoir for the development of systemic infection, is a source of methicillin-resistant strains and could pose an increased susceptibility to blood-related infections^58,59^. *Staphylococcus* further plays a sinister role in the acidic hypoxic environments of oral cancer^47^ and has been isolated from OSCC sites^60^.

Species found in abundance in the buccal microbiome of Toombak users included *Gemella morbillorum* (98.28% ident), *Prevotella nigrescens* (99.35% ident), *Staphylococcus caprae* (100% ident) and *Lachnoanaerobaculum gingivalis* (99.32% ident). *Gemella morbillorum* has been significantly associated with inflammatory gingival responses and has been detected with high abundance on the surfaces of some oral cancer tissue^60^ ^61^. It is also known to contribute to the acidic and hypoxic environments of oral cancer, promoting cancer growth^62,63^. *Gemella morbillorum* has also been the cause of infective endocarditis amongst a smokeless tobacco user from Somalia^64^. The ability of *Staphylococcus caprae* to produce B haemolysins and enterotoxins can lead to significant cell damage amongst oral cells^65^. On the other hand, species distinguishing the buccal mucosa of non-users of Toombak included *Rothia mucilaginosa* (98.88% ident), *Veillonella atypica* (98.28% ident), *Streptococcus sobrinus* (99.35% ident), *Prevotella salivae* (99.35% ident), *Prevotella pallens* (99.35% ident) and *Bifidobacterium dentium* (99.33% ident).

### The dorsum tongue mucosa

*Actinomyces* (*p* = 0.013) and uncultured bacterium (*p* = 0.031) were found to be enriched in the tongue of Toombak users. *Tannerella* (q = 0.0085) and *Cardiobacterium* (q = 0.0097) in Toombak users, were relatively abundant in this mucosal location with a mean change of 5 and 13 respectively compared to non-users*.* A *Fusobacteria* unidentified species *(OTU_1157)* was also associated with Toombak use (LDA = 2.69). In non-users, *Bifidobacterium* (q = 0.0049) was significantly increased by a mean change of 1.15 while five unique genera in the non-users of smokeless tobacco were identified; *Olsenella* (*p* = 0.04), *Lactobacillus, F0058, Aggregatibacter* (*p* = 0.02), and *Actinobacillus* (*p* = 0.01). Pattern search of the top 25 genera revealed that *Bergeyella* (*p* = 0.02), *uncultured bacteria* and *Leptotrichia (p* = *0.007)* were the most positively correlated genera associated with Toombak use, while *Scardovia (p* = *0.002)* and *Lactobacillus* were positively correlated with non-users. LEfSe plotting further highlighted that *Haemophilus* (*p* = 0.004, LDA = 3.7), *Actinobacillus* (0.003, LDA = 3.8)*,* and *Scardovia* were discriminant genera for the dorsum tongue of non-users (Fig. 7b) while *Actinomyces* distinguished the dorsum tongue of Toombak users (LDA = 3.9).

The species *Leptotrichia wadei* (q = 0.023, 99.55% ident), *Schaalia odontolytica* (*p* = 0.0028) and *Streptococcus equinus* (*p* = 0.043, 99.78% ident) were abundant in the microbiome of the dorsum tongue of Toombak users while *Haemophilus haemolyticus* (q = 0.001, 99.78% ident) and *Streptococcus sobrinus* (q = 0.023, 99.35% ident) were enriched amongst non-users of Toombak. *Corynebacterium argentoratense* (LDA = 3.7) and *Actinobacillus pleuropneumoniae* (LDA = 2.64) were also associated with the tongue of non-users.

### The floor of the mouth mucosa

Significant enrichment of the genera *Corynebacterium_1* (*p* = 0.0028), *Staphylococcus* (q = 0.01) and *Candidatus_Saccharimonas* (*p* = 0.026) was found in the floor of the mouth in those utilising Toombak while *Scardovia* (q = 0.04) was found to be high in non-users. LEfSe plotting distinguished uncultured bacterium (LDA = 3.1) *and Porphyromonas gingivalis* (LDA = 3.09) as the most distinctive microbiome components in the floor of the mouth of Toombak users (Fig. 7c). Five unique genera were present in Toombak users: *Selenomonas_4, Filifactor, Eubacterium_nodatum group, Candidatus_Saccharimonas*, *Anaeroglobus* while one genus was unique to non-users*; Catonella*. Pattern search further revealed that *Corynebacterium_1, Candidatus Saccharimonas, Stomatobaculum, Parvimonas*, and *Oribacterium* were positively correlated with Toombak use while *Prevotella_2, Lactobacillus, Scardovia, Actinobacillus, Comamonas,* and *Aggregatibacter* were the positively correlated genera with non-users of Toombak.

Species inhabiting the floor of the mouth of Toombak users included *Stomatobaculum longum, Eubacterium infirmum, Prevotella denticola,* and *Bifidobacterium dentium,* while *Corynebacterium argentoratense* was evident in the floor of the mouth of non-users.

### The hard palatal mucosa

In the palatal mucosa, positive microbiome correlations with Toombak use included *Candidatus Saccharimonus* (*p* = 0.086), *Lautropia* (q = 0.002, LDA = 3.3), *Johnsenella* (*q* = 0.001), *Actinomyces, Capnocytophaga*, *Leptotrichia*, *Faucicola* (*q* = 0.001), and *Peptococcus* while non-users of Toombak had positive correlations with *Prevotella and Prevotella_6, Selenomonas*, *Anaeroglobus*, *Lactobacillus, Moraxella, Shigella, Scardovia* (*q* = 0.012) *and Simonsiella* (*q* = 0.04). An unclassified *Gracilibacteria* oral taxon 873 (*p* = 0.031) was found to be unique to the palatal microbiome of Toombak users. Several OTUs from the *Patescibacteria* phylum were significantly abundant amongst Toombak users and included OTU_112 (*p* = 0.032), OTU_144 (*p* = 0.023), OTU_24 (*p* = 0.039), OTU_113 (*p* = 0.031) and OTU_114 (*p* = 0.043).

The species, *Leptotrichia hofstadii* was highly correlated with the hard palatal mucosa of Toombak users (99.32% ident) and *Streptococcus salivarius* was abundant. *Actinomyces israelii, Actinomyces cardiffensis* and *Faucicola mancuniensis*, were detected only in the palate of Toombak users. LEfSe plotting of species discriminant of Toombak users highlighted *Faucicola mancuniensis*, a *Bacteroidetes oral taxon 274 strain F008* and uncultured bacterium from the phyla *Fusobacteria* and *Proteobacteria* (Fig. 7d).

In comparison, *Bifidobacterium longum* (99.33% ident), *Veillonella atypica* (98.28% ident) and *Alloscardovia omnicolens* (99.33% ident) were positively correlated with the palatal mucosal microbiome of non-users while *Haemophilus paraurethrae, Haemophilus quentini,* and *Prevotella denticola* were found in non-users and *Actinomyces graevenitzii* in both groups. *Actinomyces cardiffensis* and *Actinomyces israelii* were only found on the palatal microbiome of Toombak users. Deseq 2 highlighted four OTUs abundant amongst Toombak users with q values < 0.05 that included OTU 113 (weak similarity with *Terrisporobacter*), OTU 427 (high similarity with *Neisseria* species), *Lautropia mirabilis* (BLASTn 99.35% ident) and *Leptotrichia*. *Poryphromonas cataniae* (98% ident) was significantly abundant in Toombak users.

### *Actinomyces* abundance in the saliva, tongue, buccal cheek, and hard palate of Toombak users

We found an abundance of *Actinomyces* in the saliva (q = 0.0045), dorsum tongue (*p* = 0.013), and hard palate (*p* = 0.001) of Toombak users. Reports on the effects of smokeless tobacco on *Actinomyces* growth has been contradictory. Smokeless tobacco use has been found to both reduce^66,67^ and enrich *Actinomyces* growth^68^. *Actinomyces* has also been found in abundance in moist Indian smokeless tobacco products^69^. *Actinomyces massiliensis* (*p* = 0.016) was abundant in the buccal microbiome of Toombak users while *Actinomyces graevenitzii* was found to be enriched in the tongue in both users and non-users of Toombak. *Actinomyces* is associated with the discolouration of teeth^70^ while *Actinomyces graevenitzii* has been associated with halitosis^71^. *Actinomyces graevenitzii* and *Staphylococcus* in co-culture were also found to significantly reduce neutrophil recruitment a factor that could participate in immune dysregulation amongst Toombak users^72^. Furthermore, many *Actinomyces* species are potent nitrate-reducing bacteria and thus may contribute to increasing tobacco-specific nitrosamine production in Toombak^73^. In other studies, *Actinomyces meyeri* was found to be abundant amongst smokeless tobacco users from Saudi Arabia^34^. *Actinomyces israelii* and *Actinomyces cardiffensis* were found to be unique to the palatal microbiome of Toombak users.

### *Lautropia* abundance in the saliva, buccal cheek and palate microbiomes of Toombak users could relate to compromised local immunity

In this study, *Lautropia* was abundant in the saliva, buccal cheek, and palate microbiomes of Toombak users with *Lautropia mirabilis* the species identified. *Lautropia* has been associated with benign lesions in the oral cavity such as fibroepithelial polyp^74^ as well as malignant diseases including non-small cell lung cancer, oral and oesophageal cancers^75,76^. In a study assessing Srilankan betel quid users, *Lautropia* was not abundant amongst smokeless tobacco users but rather in healthy controls^77^. In another study, *Lautropia* has been detected as part of the core oral microbiome amongst healthy Nigerians^78^, however, *Lautropia mirabilis* abundance may also be associated with compromised local immunity that could be caused by Toombak use. The *Lautropia* genus was also found to be abundant in the oral microbiome of a series of HIV-infected children^79^.

### Prevotella abundance amongst non-users of Toombak

Although *Prevotella* was present in both users and non-users, they were significantly more abundant in non-users of Toombak (*p* < 0.05) and were found in the saliva, tongue, buccal cheek and palatal microbiomes. *Alloprevotella* amongst non-users was also higher compared to Toombak users. *Prevotella* is often a commensal oral microorganism and in the oral cavity has been found to be more abundant amongst those with African and Indian heritage^80–82^. *Prevotella* abundance in the oral cavity has been associated with the development of other diseases that includes rheumatoid arthritis, metabolic syndrome, inflammatory bowel and cardiovascular disease but in other studies its abundance is associated with the reduction of hypersensitivity reactions^83,84^.

### Species modifications may harbour distinct susceptibilities in the oral cavity of Toombak users

The most common bacteria in the oral cavity were found to vary in species due to Toombak smokeless tobacco use. This may allow for a complex integration of bacteria with a more sinister ability to transfer and integrate features such as antibiotic-resistance genes eliciting distinct susceptibilities in the oral microbiome of Toombak users. Common to both users and non-users of Toombak were the species *Prevotella salivae* and *Prevotella pallens* which were found to be abundant in the tongue and palate of both groups and the buccal microbiome of non-users. They are common to the oral microbiome of adults^85^. *Prevotella scopos* and *Prevotella veroralis* were also evident in the tongue and palate microbiomes in both groups. In one study *Prevotella salivae* was increased in the buccal mucosa of smokers^86^. Although *Prevotella pallens* is commonly associated with oral health^87^, in some studies, it has been associated with development of halitosis (oral malodour) as well as OSCC^88,89^.

*Prevotella nigrescens* however was abundant only in Toombak users. Intra-orally, this species has been associated with inflammatory changes to the mucosa^90^ and alveolar bone loss^83^, while extra-orally, it has been detected in samples of occluded arteries (vascular disease)^91^. *Prevotella nigrescens* abundance did not differ in a study of betel nut users and non-users in Thailand^92^. *Prevotella nigrescens* and *Prevotella pallens* have also been associated with penicillin resistance^93^.

In the tongue, *Streptococcus sobrinus* was abundant in non-users, while *Streptococcus equinus* was abundant in Toombak users. *Streptococcus salivarius* was abundant in the hard palate of Toombak users^90^. Species from the *Streptococcus bovis/Streptococcus equinus* complex are some of the most antibiotic-resistant *Streptococcus* species found in the oral cavity^94^. It is interesting that on the dorsum tongue in Toombak users, *Streptococcus sobrinus* (a caries-associated bacterium but with no known antibiotic resistance) is replaced with *Streptococcus equinus*. *Streptococcus salivarius* found in abundance in the hard palate of Toombak users has been shown to elicit the release of the pro-inflammatory interleukin 8^95^ and is known to be a potent acetaldehyde producer^34^. Non-sucrose-containing smokeless tobacco forms, such as Toombak have also been shown to be a possible source of growth substrate for various *Streptococcus* species including *Streptococcus salivarius*^96^. Therefore, it is probable that the use of smokeless tobacco, Toombak contributes to a modified *Streptococcus* inhabitance in the oral cavity that leads to specific alterations in the various oral mucosal locations. *Streptococcus* species associated with antibiotic resistance increase in those who use Toombak compared to non-users which could be explained by the increased microbiome entry from the Toombak itself into the oral cavity as well as alterations from mechanical, chemical, and pH changes that arise from its use. *Streptococcus mitis* was the most abundant species in all sites of the oral cavity, while *Streptococcus porcorum* was abundant in the palate and tongue in both users and non-users. *Streptococcus mitis* species are regarded as commensals in the oral cavity but some strains harbour unusually high levels of resistance to β lactam antibiotics^97^. In one study, *Streptococcus mitis* was found to be abundant in those with OSCC^98^ while in another study, *Streptococcus mitis* was shown to have therapeutic potential in the treatment of oral cancer^99^.

The family*, Veillonellaceae* were found to be in similar abundance in both groups. The genus *Veillonella* is generally associated with good oral health^100^ and were relatively abundant in the tongue of both users and non-users. *Veillonella rogosae* was found on all mucosal surfaces. *Veillonella atypica* was abundant in the palate and tongue of Toombak users and non-users and the buccal cheek of non-users. In Toombak users, *Veillonella atypica* may have a role to play in nitrate/nitrite reduction from smokeless tobacco into the carcinogenic tobacco-specific nitrosamines^101^.

### Non-users of smokeless tobacco harbour genera with pro-mucosal integrity potential that include *Scardovia, Bifidobacterium* and *Lactobacillus*

Associated with healthy mucosal integrity are the genera *Scardovia*^102^, and *Lactobacillus*^56^ while *Bifidobacterium* help in the maintenance of epithelial integrity and promotion of healthy gingival barriers^103^. Interestingly, *Scardovia* is found to be depleted in oral cancer^104^ and in this study was enriched in the supragingival plaque of non-users and could distinguish the buccal mucosal microbiome of the same group. Therefore, the absence of this genus amongst Toombak users may highlight a lack of mucosal protection against oral cancer. *Lactobacillus* was enriched in the plaque of non-users of Toombak and was part of the core genera of the tongue microbiome in non-users. *Lactobacillus* was also abundant in the paraffin-embedded tissue OSCC samples taken from non-users of Toombak. *Bifidobacterium* was enriched in the plaque of non-users, *Bifidobacterium dentium* in the tongue and buccal cheek microbiome and *Bifidobacterium longum* on the hard palate of non-users.

*Lactobacillus* and *Bifidobacterium* could have many beneficial activities in the oral cavity that may be lost with Toombak use. They can produce antimicrobial compounds and have a key role in supporting the oral immune defence mechanisms. IgA levels in saliva have been shown to increase in those with *Lactobacillus* and *Bifidobacterium* abundances^105^. *Bifidobacterium dentium* is ‘mucin’ friendly preventing the disruption of the mucous barrier^106^. *Bifidobacterium* is reported to reduce DNA damage and may delay or even prevent the onset of cancers^107^. Both *Bifidobacterium* and *Lactobacillus* can help prevent the colonisation of pathogenic bacteria in the oral cavity^108^. We found *Prevotella nigrescens* for example to be abundant in Toombak users compared to non-users. *Lactobacillus* has been shown to have eliminating action against *Prevotella nigrescens* and thus its reduced presence amongst Toombak users could explain why this species was more abundant in Sudanese smokeless tobacco users^100^.

### Toombak-associated OSCC significantly carries a more aggressive microbiome

**Figure 8:**
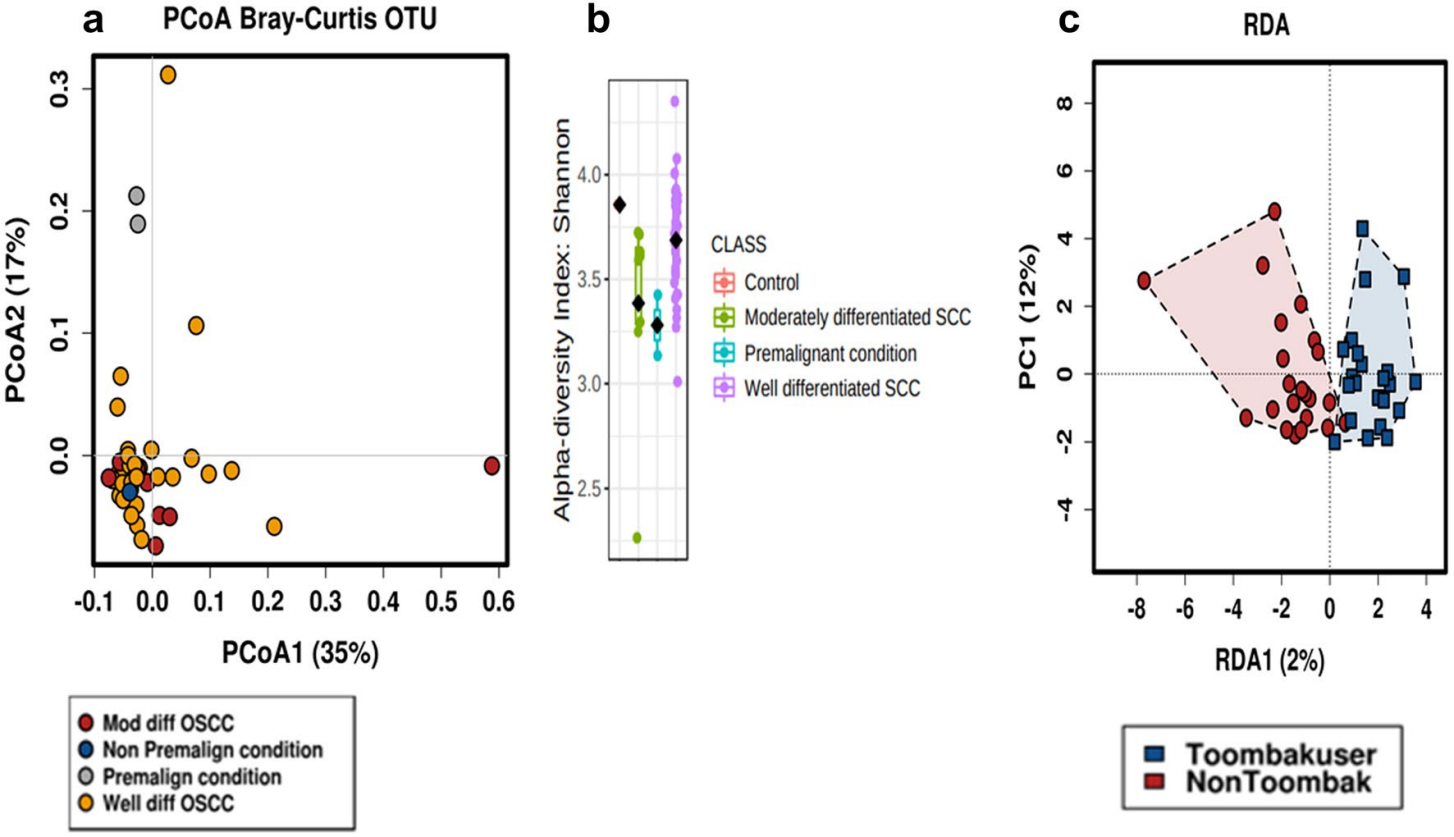
Premalignant and oral squamous cell carcinoma metagenomic profiles. (**a**) β-diversity of oral squamous cell carcinoma cancer samples from Sudanese participants utilising paraffin embedded blocks. Differentiation between premalignant and malignant samples may be observed. Malignant samples are further classified into moderate (n = 8) and well-differentiated OSCC (n = 35). (**b**) Shannon diversity (*p* = 0.03). The non-premalignant sample had the highest Shannon diversity and the premalignant samples had the lowest alpha diversity. (**c**) Redundancy analysis of oral cancer samples. Toombak use highlights microbiome variation between cancer samples compared to non-users. Oral cancer samples from non-users of Toombak had increased abundances of *Lactobacillus* (*p* = 0.024), *Collinsella* (*p* = 0.014), and *Flavobacterium* (*p* = 0.0087) while oral cancers from Toombak users had abundances in *Corynebacterium_1* (*p* = 0.044), *Mogibacterium* (*p* = 0.0013), *Enterococcus* (*p* = 0.012), *Empedobacter* (*p* = 0.03), *Stenotrophomonas* (*p* = 0.043) and *Schlegelella* (*p* = 0.048).

Clustering of the premalignant samples together could be appreciated (Fig. 8a) but β-diversity was non-significant between groups; moderate (n = 8), well-differentiated (n = 35), premalignant samples (n = 2) and the control (n = 1). It is likely, that the microbiome of moderate and well-differentiated OSCC samples is similar in microbial habitat. Shannon index between groups was statistically significant (*p* = 0.03), where the control or non-cancerous sample had the highest median alpha diversity compared to the remaining groups of oral cancer and premalignant conditions (Fig. 8b).

Redundancy analysis indicated that the oral cancer microbiome composition was influenced by Toombak use (Fig. 8c). We further applied DeSeq2 to compare mean and subsequent fold changes of the most important genera amongst Toombak and non-users of Toombak OSCC samples. OSCC from non-users of Toombak had increased abundances of *Lactobacillus* (*p* = 0.024),* Collinsella* (*p* = 0.014), and *Flavobacterium* (*p* = 0.0087) while OSCC samples from Toombak users had abundances in *Corynebacterium_1* (*p* = 0.044), *Mogibacterium* (*p* = 0.0013), *Enterococcus* (*p* = 0.012), *Empedobacter* (*p* = 0.03), *Stenotrophomonas* (*p* = 0.043) and *Schlegelella* (*p* = 0.048). *Corynebacterium_1* was significantly increased amongst those cancer samples from Toombak users (*p* = 0.044) with a reduced fold change of -1.446 amongst non–users cancer samples. Interestingly, *Corynebacterium_1* has been found to be the most abundant genus in Toombak^1^ and in this study, was found to be significantly increased in the saliva, buccal cheek and floor of the mouth microbiomes of Toombak users. *Coprococcus_2* (*p* = 0.05), *Rothia* (*p* = 1.5e−10), *Eubacterium_nodatum* (*p* = 0.0015), *Peptostreptococcus* (*p* = 6.5e−07), *Ruminiclostridium* (*p* = 0.0042) and *Stomatobaculum* (*p* = 0.013) were the most abundant genera amongst premalignant samples compared to oral cancer samples.

## Conclusion

In this study we showed that the microbiome and mycobiome of the oral cavity is significantly altered with Sudanese Toombak smokeless tobacco use. In Toombak users, non-normally residing genera become abundant throughout the hard and soft oral tissue mucosal locations that include the genus *Staphylococcus* while in non-users of Toombak, *Prevotella, Lactobacillus* and *Bifidobacterium* are found to be more prevalent. Toombak use was also found to significantly alter species determinants in genera such as *Prevotella, Streptococcus, Actinomyces* and *Veillonella.*

The mycobiome is further seen to lose counterbalance with Toombak use, with a significant enhancement of *Aspergillus* and loss of *Candida*. Such findings can pave the way for the development of ‘oral fungal and bacterial response panels’ to track OSCC progression in those with Toombak use. Further whole genomic sequencing approaches can answer how such modified communities challenge the specific functionality of the microorganism in the response to Toombak use.

While no significant local behaviours were known to otherwise alter the oral microbiome of the participants included in this study, factors such as dietary intake could play an indirect role in the host-oral microbiome connections reflected in this study requiring further research to outline other impingements on the oral microbiome of the Sudanese population.

The results of this study further contribute to a new understanding in OSCC development and its progression amongst smokeless tobacco users worldwide. We have further shown that the microbiome of Toombak related OSCC may be more aggressive resulting in an increased risk of recurrence and metastasis and an overall poorer prognosis.

## Data Availability

The datasets generated during this study are available by contacting the corresponding author upon reasonable request.

## References

[1] Sami A, Elimairi I, Patangia D, Watkins C, Ryan CA, Ross RP, Stanton C (2021). The ultra-structural, metabolomic and metagenomic characterisation of the Sudanese smokeless tobacco ‘Toombak’. Toxicol. Rep..

[2] Idris AM, Nair J, Ohshima H, Friesen M, Brouet I, Faustman EM, Bartsch H (1991). Unusually high levels of carcinogenic tobacco-specific nitrosamines in Sudan snuff (toombak). Carcinogenesis.

[3] Torti SV, Manz DH, Paul BT, Blanchette-Farra N, Torti FM (2018). Iron and cancer. Annu. Rev. Nutr..

[4] Arthur RA, Dos Santos BR, Ximenez JPB, Merlin BL, de Andrade MR, Neto JV, Fava NMN, Figueiredo DLA (2021). Microbiome and oral squamous cell carcinoma: a possible interplay on iron metabolism and its impact on tumor microenvironment. Braz. J. Microbiol..

[5] Ahmed HG (2013). Aetiology of oral cancer in the Sudan. J. Oral. Maxillofac.Res..

[6] Sajid M, Srivastava S, Joshi L, Bharadwaj M (2021). Impact of smokeless tobacco-associated bacteriome in oral carcinogenesis. Anaerobe.

[7] Buduneli N (2021). Environmental factors and periodontal microbiome. Periodontol..

[8] Willis D, Popovech M, Gany F, Zelikoff J (2012). toxicology of smokeless tobacco: implications for immune, reproductive, and cardiovascular systems. J. Toxicol. Environ. Health Part B.

[9] Alsanosy RM (2014). Smokeless tobacco (shammah) in Saudi Arabia: a review of its pattern of use, prevalence, and potential role in oral cancer. Asian Pac. J. Cancer Prev..

[10] Ibrahim SO, Vasstrand EN, Johannessen AC, Idris AM, Magnusson B, Nilsen R, Lillehaug JR (1999). Mutations of the p53 gene in oral squamous-cell carcinomas from sudanese dippers of nitrosamine-rich toombak and non-snuff-dippers from the Sudan and Scandinavia. Int. J. Cancer.

[11] Ibrahim SO, Bertelsen B, Kalvenes MB, Idris AM, Vasstrand EN, Nilsen R, Johannessen AC (1998). Expression of keratin 13, 14 and 19 in oral squamous cell carcinomas from Sudanese snuff dippers: lack of association with human papillomavirus infection. APMIS.

[12] Saxena R, Prasoodanan PKV, Gupta SV, Gupta S, Waiker P, Samaiya A, Sharma AK, Sharma VK (2022). Assessing the effect of smokeless tobacco consumption on oral microbiome in healthy and oral cancer patients. Front. Cell. Infect. Microbiol..

[13] Mohamed N, Litlekalsøy J, Ahmed IA, Martinsen EMH, Furriol J, Javier-Lopez R, Elsheikh M, Gaafar NM, Morgado L, Mundra S, Johannessen AC, Osman TA, Nginamau ES, Suleiman A, Costea DE (2021). Analysis of salivary mycobiome in a cohort of oral squamous cell carcinoma patients from Sudan identifies higher salivary carriage of malassezia as an independent and favorable predictor of overall survival. Front. Cell. Infect. Microbiol..

[14] Walsh AM, Crispie F, Kilcawley K, O’Sullivan O, O’Sullivan MG, Claesson MJ, Cotter PD (2016). Microbial succession and flavor production in the fermented dairy beverage kefir. mSystems.

[15] Magoč T, Salzberg SL (2011). FLASH: fast length adjustment of short reads to improve genome assemblies. Bioinformatics.

[16] Edgar RC (2010). Search and clustering orders of magnitude faster than BLAST. Bioinformatics.

[17] Caporaso JG, Bittinger K, Bushman FD, DeSantis TZ, Andersen GL, Knight R (2010). PyNAST: a flexible tool for aligning sequences to a template alignment. Bioinformatics.

[18] Quast C, Pruesse E, Yilmaz P, Gerken J, Schweer T, Yarza P, Peplies J, Glöckner FO (2013). The SILVA ribosomal RNA gene database project: improved data processing and web-based tools. Nucleic Acids Res..

[19] McGinnis S, Madden TL (2004). BLAST: at the core of a powerful and diverse set of sequence analysis tools. Nucleic Acids Res..

[20] Callahan BJ, McMurdie PJ, Rosen MJ, Han AW, Johnson AJA, Holmes SP (2016). DADA2: high-resolution sample inference from Illumina amplicon data. Nat. Methods.

[21] Team, R. C. R: A language and environment for statistical computing. MSOR connections 1. (2014).

[22] Martin, M. CUTADAPT removes adapter sequences from high-throughput sequencing reads. EMBnetjournal **17** (2011).

[23] Dhariwal A, Chong J, Habib S, King IL, Agellon LB, Xia J (2017). MicrobiomeAnalyst: a web-based tool for comprehensive statistical, visual and meta-analysis of microbiome data. Nucleic Acids Res..

[24] Kovács L, Kézér FL, Bakony M, Hufnágel L, Tőzsér J, Jurkovich V (2015). Associations between heart rate variability parameters and housing- and individual-related variables in dairy cows using canonical correspondence analysis. PLoS One.

[25] Harrandah AM, Chukkapalli SS, Bhattacharyya I, Progulske-Fox A, Chan EKL (2020). Fusobacteria modulate oral carcinogenesis and promote cancer progression. J. Oral Microbiol..

[26] Verma D, Garg PK, Dubey AK (2018). Insights into the human oral microbiome. Arch. Microbiol..

[27] Fujiwara N, Kitamura N, Yoshida K, Yamamoto T, Ozaki K, Kudo Y (2020). Involvement of fusobacterium species in oral cancer progression: a literature review including other types of cancer. Int. J. Mol. Sci..

[28] Espinoza JL, Harkins DM, Torralba M, Gomez A, Highlander SK, Jones MB, Leong P, Saffery R, Bockmann M, Kuelbs C, Inman JM, Hughes T, Craig JM, Nelson KE, Dupont CL, Relman DA (2018). Supragingival plaque microbiome ecology and functional potential in the context of health and disease. mBio.

[29] Sami A, Elimairi I, Patangia D, Watkins C, Ryan CA, Ross RP, Stanton C (2021). The ultra-structural, metabolomic and metagenomic characterisation of the Sudanese smokeless tobacco 'Toombak'. Toxicol. Rep..

[30] Rands CM, Starikova EV, Brüssow H, Kriventseva EV, Govorun VM, Zdobnov EM (2018). ACI-1 beta-lactamase is widespread across human gut microbiomes in Negativicutes due to transposons harboured by tailed prophages. Environ. Microbiol..

[31] Gopinath D, Kunnath Menon R, Chun Wie C, Banerjee M, Panda S, Mandal D, Behera PK, Roychoudhury S, Kheur S, George Botelho M, Johnson NW (2021). Salivary bacterial shifts in oral leukoplakia resemble the dysbiotic oral cancer bacteriome. J. Oral Microbiol..

[32] Waite DW, Chuvochina M, Pelikan C, Parks DH, Yilmaz P, Wagner M, Loy A, Naganuma T, Nakai R, Whitman WB, Hahn MW, Kuever J, Hugenholtz P (2020). Proposal to reclassify the proteobacterial classes Deltaproteobacteria and Oligoflexia, and the phylum Thermodesulfobacteria into four phyla reflecting major functional capabilities. Int. J. Syst. Evol. Microbiol..

[33] Campbell A, Campbell J, Schwientek P, Woyke T, Sczyrba A, Allman S, Beall C, Griffen A, Leys E, Podar M (2013). Multiple single-cell genomes provide insight into functions of uncultured deltaproteobacteria in the human oral cavity. PLoS One.

[34] Halboub E, Al-Akhali MS, Alamir AH, Homeida HE, Baraniya D, Chen T, Al-Hebshi NN (2020). Tongue microbiome of smokeless tobacco users. BMC Microbiol..

[35] Hernandez BY, Zhu X, Goodman MT, Gatewood R, Mendiola P, Quinata K, Paulino YC (2017). Betel nut chewing, oral premalignant lesions, and the oral microbiome. PLoS One.

[36] Chattopadhyay I, Verma M, Panda M (2019). Role of oral microbiome signatures in diagnosis and prognosis of oral cancer. Technol. Cancer Res. Treat..

[37] Pushalkar S, Ji X, Li Y, Estilo C, Yegnanarayana R, Singh B, Li X, Saxena D (2012). Comparison of oral microbiota in tumor and non-tumor tissues of patients with oral squamous cell carcinoma. BMC Microbiol..

[38] Ali MABM (2014). Isolation and Identification of Some Oral Microorganisms from healthy Sudanese Smokers and Oral Cancer Patients.

[39] Okada N, Murakami A, Sato M, Nakamura S, Fujii S, Sogabe K, Takahashi M, Okada A, Abe A, Fujii H, Abe M, Azuma M, Ishizawa K (2022). First reported case of Lachnoanaerobaculum gingivalis bacteremia in an acute myeloid leukemia patient with oral mucositis during high dose chemotherapy. Anaerobe.

[40] Sawant, S., Dugad, J., Parikh, D., Srinivasan, S. & Singh, H. Identification & correlation of bacterial diversity in oral cancer and long-term tobacco chewers- A case-control pilot study. *J. Med. Microbiol.* **70** (2021). 10.1099/jmm.0.001417 34553683

[41] Sarkar A, Stoneking M, Nandineni MR (2017). Unraveling the human salivary microbiome diversity in Indian populations. PLoS One.

[42] Al-Marzooq F, Al Kawas S, Rahman B, Shearston JA, Saad H, Benzina D, Weitzman M (2022). Supragingival microbiome alternations as a consequence of smoking different tobacco types and its relation to dental caries. Sci. Rep..

[43] Amer, A., Galvin, S., Healy, C. M., Moran, G. P. The microbiome of potentially malignant oral leukoplakia exhibits enrichment for fusobacterium, leptotrichia, campylobacter, and Rothia species. *Front. Microbiol.* **8** (2017). 10.3389/fmicb.2017.02391 PMC5717034 29250055

[44] Torres PJ, Fletcher EM, Gibbons SM, Bouvet M, Doran KS, Kelley ST (2015). Characterization of the salivary microbiome in patients with pancreatic cancer. PeerJ..

[45] Mougeot JC, Beckman MF, Langdon HC, Lalla RV, Brennan MT, Bahrani Mougeot FK (2021). *Haemophilus pittmaniae* and *Leptotrichia* spp. constitute a multi-marker signature in a cohort of human papillomavirus-positive head and neck cancer patients. Front. Microbiol..

[46] Al Bataineh MT, Dash NR, Elkhazendar M, DaMH A, Darwish IMI, Al-Hajjaj MS, Hamid Q (2020). Revealing oral microbiota composition and functionality associated with heavy cigarette smoking. J. Transl. Med..

[47] Srivastava A, Mishra S, Garg PK, Dubey AK, Deo SVS, Verma D (2022). Comparative and analytical characterization of the oral bacteriome of smokeless tobacco users with oral squamous cell carcinoma. Appl. Microbiol. Biotechnol..

[48] Proctor DM, Fukuyama JA, Loomer PM, Armitage GC, Lee SA, Davis NM, Ryder MI, Holmes SP, Relman DA (2018). A spatial gradient of bacterial diversity in the human oral cavity shaped by salivary flow. Nat. Commun..

[49] Magnussen A, Parsi MA (2013). Aflatoxins, hepatocellular carcinoma and public health. World J. Gastroenterol..

[50] Li Y, Wang K, Zhang B, Tu Q, Yao Y, Cui B, Ren B, He J, Shen X, Van Nostrand JD, Zhou J, Shi W, Xiao L, Lu C, Zhou X (2019). Salivary mycobiome dysbiosis and its potential impact on bacteriome shifts and host immunity in oral lichen planus. Int. J. Oral. Sci..

[51] Pavia CS, Plummer MM (2020). Clinical implications of nicotine as an antimicrobial agent and immune modulator. Biomed. Pharmacother..

[52] da Costa KR, Ferreira JC, Komesu MC, Candido RC (2009). Candida albicans and Candida tropicalis in oral candidosis: quantitative analysis, exoenzyme activity, and antifungal drug sensitivity. Mycopathologia.

[53] Deepa A, Nair BJ, Sivakumar T, Joseph AP (2014). Uncommon opportunistic fungal infections of oral cavity: a review. J. Oral Maxillofac. Pathol..

[54] Sankari SL, Mahalakshmi K, Kumar VN (2020). A comparative study of Candida species diversity among patients with oral squamous cell carcinoma and oral potentially malignant disorders. BMC Res. Notes.

[55] Srivastava A, Mishra SD, Garg PK, Dubey AK, Deo SVS, Verma D (2022). Comparative and analytical characterization of the oral bacteriome of smokeless tobacco users with oral squamous cell carcinoma. Appl. Microbiol. Biotechnol..

[56] Laheij AMGA, Raber-Durlacher JE, Koppelmans RGA, Huysmans M-CDNJM, Potting C, van Leeuwen SJM, Hazenberg MD, Brennan MT, von Bültzingslöwen I, Johansson J-E, de Soet JJ, Haverman TM, Buijs MJ, Brandt BW, Rozema FR, Blijlevens NMA, Zaura E (2019). Microbial changes in relation to oral mucositis in autologous hematopoietic stem cell transplantation recipients. Sci. Rep..

[57] Carolus H, Van Dyck K, Van Dijck P (2019). *Candida albicans* and Staphylococcus species: a threatening twosome. Front. Microbiol..

[58] McCormack MG, Smith AJ, Akram AN, Jackson M, Robertson D, Edwards G (2015). Staphylococcus aureus and the oral cavity: An overlooked source of carriage and infection?. Am. J. Infect. Control.

[59] Pasman R, Krom BP, Zaat SAJ, Brul S (2022). The role of the oral immune system in oropharyngeal candidiasis-facilitated invasion and dissemination of *Staphylococcus aureus*. Front. Oral. Health.

[60] Minarovits J (2021). Anaerobic bacterial communities associated with oral carcinoma: intratumoral, surface-biofilm and salivary microbiota. Anaerobe.

[61] Rai AK, Panda M, Das AK, Rahman T, Das R, Das K, Sarma A, Kataki AC, Chattopadhyay I (2021). Dysbiosis of salivary microbiome and cytokines influence oral squamous cell carcinoma through inflammation. Arch. Microbiol..

[62] Hettmann, A., Demcsák, A., Decsi, G., Bach, Á., Pálinkó, D., Rovó, L., Nagy, K., Takács, M. & Minarovits, J. Infectious agents associated with head and neck carcinomas. *Adv. Microbiol. Infectious Dis. Public Health* 63–80 (2015). 10.1007/5584_2015_5005 26563307

[63] Lee A, Ghaname CB, Braun TM, Sugai JV, Teles RP, Loesche WJ, Kornman KS, Giannobile WV, Kinney JS (2012). Bacterial and salivary biomarkers predict the gingival inflammatory profile. J. Periodontol..

[64] Massoure PL, Lions C, Caumes JL, Spadoni S, Gaillard PE, Bougere J (2010). Lethal aortic endocarditis due to *Gemella morbillorum* in a Djiboutian khat user. Rev. Med. Intern..

[65] Fowoyo PT, Ogunbanwo ST (2016). Virulence and toxigenicity of coagulase-negative staphylococci in Nigerian traditional fermented foods. Can. J. Microbiol..

[66] Bhatt S, Rajesh G, Pai MB, Rao A, Shenoy R (2014). Effects of refined extracts of smokeless tobacco on Cariogenic microorganisms: an in-vitro study. Int. J. Adv. Res..

[67] Liu M, Jin J, Pan H, Feng J, Cerniglia CE, Yang M, Chen H (2016). Effect of smokeless tobacco products on human oral bacteria growth and viability. Anaerobe.

[68] Vishwakarma A, Verma D (2021). Microorganisms: crucial players of smokeless tobacco for several health attributes. Appl. Microbiol. Biotechnol..

[69] Sajid M, Srivastava S, Kumar A, Kumar A, Singh H, Bharadwaj M (2021). Bacteriome of moist smokeless tobacco products consumed in India with emphasis on the predictive functional potential. Front. Microbiol..

[70] Mortazavi, H., Baharvand, M. & Khodadoustan, A. Colors in tooth discoloration: a new classification and literature review. *Int. J. Clin. Dentistry* **7** (2014).

[71] Bernardi S, Karygianni L, Filippi A, Anderson AC, Zürcher A, Hellwig E, Vach K, Macchiarelli G, Al-Ahmad A (2020). Combining culture and culture-independent methods reveals new microbial composition of halitosis patients' tongue biofilm. MicrobiologyOpen.

[72] Jalali F, Ellett F, Balani P, Duncan MJ, Dewhirst FE, Borisy GG, Irimia D (2021). No man's land: species-specific formation of exclusion zones bordering *Actinomyces graevenitzii* microcolonies in nanoliter cultures. MicrobiologyOpen.

[73] Gordon JH, LaMonte MJ, Genco RJ, Zhao J, Li L, Hovey KM, Tsompana M, Buck MJ, Andrews CA, Mcskimming DI (2019). Is the oral microbiome associated with blood pressure in older women?. High Blood Pressure Cardiovasc. Prevent..

[74] Perera M, Al-Hebshi N, Perera I, Ipe D, Ulett G, Speicher D, Chen T, Johnson N (2018). Inflammatory bacteriome and oral squamous cell carcinoma. J. Dent. Res..

[75] Zhang W, Luo J, Dong X, Zhao S, Hao Y, Peng C, Shi H, Zhou Y, Shan L, Sun Q (2019). Salivary microbial dysbiosis is associated with systemic inflammatory markers and predicted oral metabolites in non-small cell lung cancer patients. J. Cancer.

[76] Schmidt BL, Kuczynski J, Bhattacharya A, Huey B, Corby PM, Queiroz EL, Nightingale K, Kerr AR, DeLacure MD, Veeramachaneni R, Olshen AB, Albertson DG (2014). Changes in abundance of oral microbiota associated with oral cancer. PLoS One.

[77] Uehara O, Hiraki D, Kuramitsu Y, Matsuoka H, Takai R, Fujita M, Harada F, Paudel D, Takahashi S, Yoshida K, Muthumala M, Nagayasu H, Chiba I, Abiko Y (2021). Alteration of oral flora in betel quid chewers in Sri Lanka. J. Microbiol. Immunol. Infect..

[78] Anukam KC, Onwuzor I, Olise N, Duru M, Agbakoba N (2018). Oral bacteriome compositions identified by 16S rRNA metagenomics in a randomly selected “healthy” Nigerian male and female subjects. Int. J. Res. Rep. Dentistry.

[79] Rossmann SN, Wilson PH, Hicks J, Carter B, Cron SG, Simon C, Flaitz CM, Demmler GJ, Shearer WT, Kline MW (1998). Isolation of <i>*Lautropia mirabilis*</i> from oral cavities of human immunodeficiency virus-infected children. J. Clin. Microbiol..

[80] Yang Y, Zheng W, Cai Q, Shrubsole MJ, Pei Z, Brucker R, Steinwandel M, Bordenstein SR, Li Z, Blot WJ, Shu X-O, Long J, Xu J (2019). Racial differences in the oral microbiome: data from low-income populations of African ancestry and European ancestry. mSystems.

[81] Downes J, Hooper SJ, Wilson MJ, Wade WG (2008). *Prevotella histicola* sp. nov., isolated from the human oral cavity. Int. J. Syst. Evol. Microbiol..

[82] Acharya A, Chan Y, Kheur S, Kheur M, Gopalakrishnan D, Watt R, Mattheos N (2017). Salivary microbiome of an urban Indian cohort and patterns linked to subclinical inflammation. Oral Dis..

[83] Larsen JM (2017). The immune response to Prevotella bacteria in chronic inflammatory disease. Immunology.

[84] Murugesan S, Elanbari M, Bangarusamy DK, Terranegra A, Al KS (2021). Can the salivary microbiome predict cardiovascular diseases? Lessons learned from the Qatari population. Front. Microbiol..

[85] Könönen E, Gursoy UK (2021). Oral Prevotella species and their connection to events of clinical relevance in gastrointestinal and respiratory tracts. Front. Microbiol..

[86] Karabudak S, Ari O, Durmaz B, Dal T, Basyigit T, Kalcioglu MT, Durmaz R (2019). Analysis of the effect of smoking on the buccal microbiome using next-generation sequencing technology. J. Med. Microbiol..

[87] Fteita D, Könönen E, Gürsoy M, Ma X, Sintim HO, Gürsoy UK (2018). Quorum sensing molecules regulate epithelial cytokine response and biofilm-related virulence of three Prevotella species. Anaerobe.

[88] Jia YJ, Liao Y, He YQ, Zheng MQ, Tong XT, Xue WQ, Zhang JB, Yuan LL, Zhang WL, Jia WH (2021). Association between oral microbiota and cigarette smoking in the Chinese population. Front. Cell Infect. Microbiol..

[89] Riggio MP, Lennon A, Rolph HJ, Hodge PJ, Donaldson A, Maxwell AJ, Bagg J (2008). Molecular identification of bacteria on the tongue dorsum of subjects with and without halitosis. Oral Dis..

[90] Saxena R, Prasoodanan PKV, Gupta SV, Gupta S, Waiker P, Samaiya A, Sharma AK, Sharma VK (2022). Assessing the effect of smokeless tobacco consumption on oral microbiome in healthy and oral cancer patients. Front. Cell Infect. Microbiol..

[91] Iwai T, Inoue Y, Umeda M, Huang Y, Kurihara N, Koike M, Ishikawa I (2005). Oral bacteria in the occluded arteries of patients with Buerger disease. J. Vasc. Surg..

[92] Dahlén G, Nauclér C, Nordwall S, Suksu-art N (2010). Oral microflora in betel-chewing adults of the Karen tribe in Thailand. Anaerobe.

[93] Mättö J, Asikainen S, Väisänen ML, Von Troil-Lindén B, Könönen E, Saarela M, Salminen K, Finegold SM, Jousimies-Somer H (1999). Beta-lactamase production in *Prevotella intermedia*, *Prevotella nigrescens*, and *Prevotella pallens* genotypes and in vitro susceptibilities to selected antimicrobial agents. Antimicrob. Agents Chemother..

[94] Pompilio A, Di Bonaventura G, Gherardi G (2019). An overview on *Streptococcus bovis/Streptococcus equinus* complex isolates: identification to the species/subspecies level and antibiotic resistance. Int. J. Mol. Sci..

[95] Mostefaoui Y, Bart C, Frenette M, Rouabhia M (2004). *Candida albicans* and *Streptococcus salivarius* modulate IL-6, IL-8, and TNF-α expression and secretion by engineered human oral mucosa cells. Cell. Microbiol..

[96] Vellappally S, Fiala Z, Šmejkalová J, Jacob V, Shriharsha P (2007). Influence of tobacco use in dental caries development. Central Eur. J. Public Health.

[97] König A, Reinert RR, Hakenbeck R (1998). *Streptococcus mitis* with unusually high level resistance to β-lactam antibiotics. Microb. Drug Resistance.

[98] Mager DL, Haffajee AD, Devlin PM, Norris CM, Posner MR, Goodson JM (2005). The salivary microbiota as a diagnostic indicator of oral cancer: a descriptive, non-randomized study of cancer-free and oral squamous cell carcinoma subjects. J. Transl. Med..

[99] Baraniya D, Jain V, Lucarelli R, Tam V, Vanderveer L, Puri S, Yang M, Al-Hebshi NN (2020). Screening of health-associated oral bacteria for anticancer properties in vitro. Front. Cell. Infect. Microbiol..

[100] Hojo K, Mizoguchi C, Taketomo N, Ohshima T, Gomi K, Arai T, Maeda N (2007). Distribution of salivary Lactobacillus and Bifidobacterium species in periodontal health and disease. Biosci. Biotechnol. Biochem..

[101] Doel JJ, Benjamin N, Hector MP, Rogers M, Allaker RP (2005). Evaluation of bacterial nitrate reduction in the human oral cavity. Eur. J. Oral Sci..

[102] McDaniel S, McDaniel J, Howard KM, Kingsley K (2021). Molecular screening and analysis reveal novel oral site-specific locations for the cariogenic pathogen *Scardovia wiggsiae*. Dentistry J..

[103] Takahashi N, Sulijaya B, Yamada-Hara M, Tsuzuno T, Tabeta K, Yamazaki K (2019). Gingival epithelial barrier: regulation by beneficial and harmful microbes. Tissue Barriers.

[104] Börnigen D, Ren B, Pickard R, Li J, Ozer E, Hartmann EM, Xiao W, Tickle T, Rider J, Gevers D, Franzosa EA, Davey ME, Gillison ML, Huttenhower C (2017). Alterations in oral bacterial communities are associated with risk factors for oral and oropharyngeal cancer. Sci. Rep..

[105] Lin CW, Chen YT, Ho HH, Hsieh PS, Kuo YW, Lin JH, Liu CR, Huang YF, Chen CW, Hsu CH, Lin WY, Yang SF (2021). Lozenges with probiotic strains enhance oral immune response and health. Oral Dis..

[106] Engevik MA, Luk B, Chang-Graham AL, Hall A, Herrmann B, Ruan W, Endres BT, Shi Z, Garey KW, Hyser JM, Versalovic J (2019). Bifidobacterium dentium fortifies the intestinal mucus layer via autophagy and calcium signaling pathways. mBio.

[107] Grover HS, Luthra S (2011). Probiotics–the nano soldiers of oral health. J. Indian Acad. Clin. Med..

[108] Meurman JH (2005). Probiotics: Do they have a role in oral medicine and dentistry?. Eur. J. Oral Sci..

